# Supplementary material for: Supervised treatment in outpatients for schizophrenia plus (STOPS+): protocol for a cluster randomised trial of a community-based intervention to improve treatment adherence and reduce the treatment gap for schizophrenia in Pakistan
Source: BMJ Open. 2020 Jun 30;10(6):e034709. doi: 10.1136/bmjopen-2019-034709 (PMC7328742; doi:10.1136/bmjopen-2019-034709)
Supplement: Author's manuscript [file bmjopen-2019-034709.draft_revisions.pdf]

BMJ Open is committed to open peer review. As part of this commitment we make the peer review history of every article we publish publicly available.

When an article is published we post the peer reviewers' comments and the authors' responses online. We also post the versions of the paper that were used during peer review. These are the versions that the peer review comments apply to.

The versions of the paper that follow are the versions that were submitted during the peer review process. They are not the versions of record or the final published versions. They should not be cited or distributed as the published version of this manuscript.

BMJ Open is an open access journal and the full, final, typeset and author-corrected version of record of the manuscript is available on our site with no access controls, subscription charges or pay-per-view fees (http://bmjopen.bmj.com).

If you have any questions on BMJ Open's open peer review process please email info.bmjopen@bmj.com

BMJ Open

Supervised Treatment in Outpatients for Schizophrenia Plus (STOPS+): Protocol for a cluster randomised trial of a community-based intervention to improve treatment adherence and reduce the treatment gap for schizophrenia in Pakistan

Journal:	*BMJ Open*
Manuscript ID	bmjopen-2019-034709
Article Type:	Protocol
Date Submitted by the Author:	02-Oct-2019

Complete List of Authors:	Shepherd, Thomas; Keele University, School of Primary, Community and Social Care<br>Ul-Haq, Zia; Khyber Medical University, Institute of Public Health & Social Sciences<br>Ul-Haq, Mian; Lady Reading Hospital<br>Khan, Muhammad Firaz; Lady Reading Hospital<br>Afridi, Adil; Lady Reading Hospital<br>Dikomitis, Lisa; Keele University, School of Primary, Community and Social Care; Keele University, School of Medicine<br>Robinson, Michelle; Keele University, School of Primary, Community and Social Care<br>Lewis, Martyn; Keele University, School of Primary, Community and Social Care<br>Rahman, Atif; University of Liverpool, Child Mental Health Unit<br>Dziedzic, Krysia; Keele University, School of Primary, Community and Social Care<br>Saeed, Umaima; Khyber Medical University, Institute of Public Health & Social Sciences<br>Awan, Naila; Lady Reading Hospital<br>Mallen, Christian; Keele University, School of Primary, Community and Social Care; Midlands Partnership Foundation Trust<br>Farooq, Saeed; Keele University, School of Primary, Community and Social Care; Midlands Partnership Foundation Trust

Keywords:	Schizophrenia & psychotic disorders < PSYCHIATRY, MENTAL HEALTH, Clinical trials < THERAPEUTICS, PUBLIC HEALTH

SCHOLARONE™
Manuscripts

I, the Submitting Author has the right to grant and does grant on behalf of all authors of the Work (as defined in the below author licence), an exclusive licence and/or a non-exclusive licence for contributions from authors who are: i) UK Crown employees; ii) where BMJ has agreed a CC-BY licence shall apply, and/or iii) in accordance with the terms applicable for US Federal Government officers or employees acting as part of their official duties; on a worldwide, perpetual, irrevocable, royalty-free basis to BMJ Publishing Group Ltd ("BMJ") its licensees and where the relevant Journal is co-owned by BMJ to the co-owners of the Journal, to publish the Work in this journal and any other BMJ products and to exploit all rights, as set out in our licence.

The Submitting Author accepts and understands that any supply made under these terms is made by BMJ to the Submitting Author unless you are acting as an employee on behalf of your employer or a postgraduate student of an affiliated institution which is paying any applicable article publishing charge ("APC") for Open Access articles. Where the Submitting Author wishes to make the Work available on an Open Access basis (and intends to pay the relevant APC), the terms of reuse of such Open Access shall be governed by a Creative Commons licence – details of these licences and which Creative Commons licence will apply to this Work are set out in our licence referred to above.

Other than as permitted in any relevant BMJ Author's Self Archiving Policies, I confirm this Work has not been accepted for publication elsewhere, is not being considered for publication elsewhere and does not duplicate material already published. I confirm all authors consent to publication of this Work and authorise the granting of this licence.

For peer review only

Supervised Treatment in Outpatients for Schizophrenia Plus (STOPS+): Protocol for a cluster randomised trial of a community-based intervention to improve treatment adherence and reduce the treatment gap for schizophrenia in Pakistan

Thomas A. Shepherd[1], Zia Ul Haq[2], Mian Muktar ul-Haq[3], Muhammad Firaz Khan[3], Adil Afridi[3], Lisa Dikomitis[1.6], Michelle E. Robinson[1], Martyn Lewis[1], Atif Rahman[4], , Krysia Dziedzic[1], Umaima Saeed[2], Naila Riaz Awan[3] Christian D. Mallen[1, 5] & Saeed Farooq[1,5]

[1]School of Primary, Community and Social Care, Keele University, Staffordshire, ST5 5BG, UK

[2]Khyber Medical University, Phase V, Hayatabad, Peshawar, Khyber Pakhtunkhwa, 25100 Pakistan

[3]Lady Reading Hospital, Soekarno Rd, Pipal Mandi, Peshawar, Khyber Pakhtunkhwa 25000, Pakistan

[4]University of Liverpool, Foundation Building, Brownlow Hill, Liverpool, L69 7TX, UK

[5]Midlands Partnership Foundation Trust, Trust Headquarters, Trust Head Quaters Mellor House, Corporation St, Stafford ST16 3SR

[6] School of Medicine, Keele University Staffordshire, ST5 5BG, UK

Correspondence: Prof. Saeed Farooq, Faculty of Medicine and Health Sciences, Keele University, [1]School of Primary, Community and Social Care, Keele University, Staffordshire, ST5 5BG, UK

E mail: s.farooq@keele.ac.uk
Telephone: 01782 734973

Keywords: Psychosis, Low and middle Income Countries (LMIC), Drug Treatment, Directly Observed Therapy (DOTS)

Word Count: 4690

For peer review only - http://bmjopen.bmj.com/site/about/guidelines.xhtml

ABSTRACT

Introduction: There is a significant treatment gap, with only a few community-based services for people with schizophrenia in Low- and Middle-Income countries. Poor treatment adherence in schizophrenia is associated with poorer health outcomes, suicide attempts and death. We previously reported the effectiveness of STOPS (Supervised Treatment in Outpatients for Schizophrenia) for improving treatment adherence in patients with schizophrenia. However, STOPS was evaluated in a tertiary care setting with no primary care involvement, limiting its generalisability to the wider at-risk population. We aim to evaluate the effectiveness of STOPS+ in scaling up the primary care treatment of schizophrenia to a real-world setting.
Methods: The effectiveness of the STOPS+ intervention in improving the level of functioning and medication adherence in patients with schizophrenia in Pakistan will be evaluated using a cluster randomised controlled trial design. We aim to recruit 526 participants from 24 Primary Health Care Centres (PHCs) randomly allocated in 1;1 ratio to STOPS+ intervention and Enhanced Treatment as Usual (ETAU) arms. Participants will be followed-up for 12 months' post recruitment.
Analysis: The sample size is estimated for two outcomes (i) The primary clinical outcome is level of functioning, measured using the Global Assessment of Functioning (GAF) scale, and (ii) the primary process outcome is adherence to treatment regimen measured using a validated measure. An intention-to-treat approach will be used for the primary analysis.
Ethics and Dissemination: Ethical approval has been obtained from Keele University Ethical Review Panel (ref: MH-190017) and Khyber Medical University Ethical Review Board (ref: DIR-KMU-EB/ST/000648). The results of the STOPS+ trial will be reported in peer reviewed journals and academic conferences and disseminated to local stakeholders and policy makers.

Trial Registration information: https://doi.org/10.1186/ISRCTN93243890

For peer review only

STRENGTHS AND LIMITATIONS OF THIS STUDY

- This study aims to evaluate an approach that can be used for scaling up treatment for schizophrenia in resource poor settings and specifically addresses the gap in schizophrenia treatment.
- To the best of our knowledge this is first study that uses a DOTS (Directly Observed Therapy) approach translated from an infectious disorder i.e. Tuberculosis to a non-communicable disorder for scaling up the treatment for schizophrenia.
- The intervention involves primary care in treatment of schizophrenia in community, which is rarely done in LMIC.
- The trial is powered two primary outcomes (i) a clinical outcome measure – Global Assessment of Functioning and (ii) a process outcome measure – adherence to medication.
- The measurement of treatment adherence is based on a subjective measure i.e. reporting from patients and carers that cannot be objectively validated.

For peer review only

INTRODUCTION

Schizophrenia one of the leading causes of years lived with disability, especially in low- and middle-income countries (LMICs) [1]. Existing evidence-based interventions for schizophrenia have been poorly implemented, with only 31% of people with schizophrenia accessing treatment in low- and middle-income countries (LMIC).[2] For instance, Pakistan, with a population of about 200 million, has one of the largest treatment gaps for schizophrenia amongst LMIC.[3]

This treatment gap is attributed to a number of factors including poor treatment adherence with average non treatment adherence rates of about 50% in schizophrenia,[4] a lack of primary care involvement and little to no access to treatment.[5] Whilst the cost of antipsychotic medication in Pakistan is relatively low, economic and logistical factors remain significant barriers to medication access [6] and the purchase of antipsychotic medication is often at the expense of other forms of essential out-of-pocket healthcare or even food.[7]

Reducing the treatment gap for schizophrenia in LMIC is critical. Medication non-adherence in patients with schizophrenia is associated with 2.8 times increased risk in hospital admission.[8] The risk of death in patients who receive even minimal treatment is significantly reduced compared with patients receiving no treatment at all.[9] Furthermore, untreated psychosis can at least double the costs of managing chronic co-morbid physical health conditions, leading to an increased stress on an already overburdened healthcare system.[10]

We previously reported a randomised controlled trial (RCT) of Supervised Treatment in Out-Patients for Schizophrenia (STOPS) in Peshawar, Pakistan. The STOPS significantly improved treatment adherence and functioning in people with schizophrenia.[11] STOPS was developed using the Medical Research Council framework for complex interventions [12] and was modelled on the Directly Observed Treatment Short-Course (DOTS) intervention to improve treatment adherence for people with Tuberculosis (TB). Following the same principles as the DOTS approach, a close relative supervised and administrated all schizophrenia medication and reported this to a health worker.

The original STOPS intervention was evaluated in a tertiary care setting by mental health professionals, with no primary care involvement, limiting its scaling up and generalisability. Through a programme of qualitative work with key stakeholders in Pakistan, including patients, carers, healthcare professionals and researchers, we adapted the STOPS model to STOPS+ with the aim of scaling up the intervention and involving primary care in the management of schizophrenia.

Here, we outline the protocol for evaluating the STOPS+ in a real world setting using a cluster RCT design.

For peer review only

METHODS AND ANALYSIS

Objectives

Primary objective

The primary objective is to evaluate the clinical effectiveness of the STOPS+ intervention in improving medication adherence and functioning in patients with schizophrenia compared to enhanced treatment as usual (ETAU) in primary care in Pakistan.

Secondary objectives

i) To evaluate the cost effectiveness of the STOPS+ intervention compared to ETAU;
ii) To assess the effectiveness of STOPS+ in reducing family/caregiver burden and stigma in the community;
iii) To evaluate if STOPS+ leads to physical health improvement in patients with schizophrenia;
iv) To investigate the implementation of STOPS+ in a primary care setting and the acceptability of STOPS+ for service users and healthcare providers, assessing the impact of STOPS+ in the wider healthcare system.

Study setting

The intervention will be implemented and evaluated in the Peshawar district of Khyber Pakhtunkhwa (KP). KP is one of four provinces in Pakistan located in the North West region with an estimated population of 20.7 million. Health expenditure in Pakistan is about 1% of the Gross Domestic Product (GDP) with no separate provision for mental health services. The primary health care (PHC) facilities in KP are provided by a network of Basic Health Units (BHUs), Civil Dispensaries (CDs), and Rural Health Centres (RHCs). Typically, each PHC facility consists of a primary care physician, a Multipurpose PHC technician (MT) and a Lady Health Worker (LHW).[13] Training PHC providers to manage mental health conditions and the incorporation of mental health services into primary care are both envisaged in current health policy, but have not yet been fully realised.[5]

Trial design

A cluster randomised controlled trial (RCT) will be used to investigate the efficacy of the STOPS+ intervention compared with Enhanced Treatment as Usual (ETAU) in people living with Schizophrenia. The cluster RCT design will help to evaluate the implementation of STOPS+ at the health care facility level and minimise the contamination of intervention and control arms.

INTERVENTION AND CONTROL

The control: Enhanced Treatment as Usual (ETAU)

Typically, in Pakistan, treatment for schizophrenia consists of accessing hospital based psychiatric services with little to no involvement of PHC and a limited provision of medication

For peer review only

from the hospital pharmacy. ETAU provided in the control clusters will include the treatment received by patients' routine health care setting, which includes treatment provided by a psychiatrist in the outpatients of one of the psychiatry department of the local hospital and brief counselling about the treatment and outcome of the disorder. Patients may be admitted to an inpatient unit at the hospital. This will be enhanced in the control cluster PHCs with training provided to PHC physicians in WHO Mental Health Gap Action Programme (mhGAP). (https://www.who.int/mental_health/mhgap/en/t) in addition to the regular and reliable provision of psychotropic drugs at the PHC. The patients will be informed that the antipsychotic medication is available in the PHC centre and they can assess the centre for supply of medication. The PHC physician will have the option to liaise with the treating psychiatrist, as provided in the MhGAP guidelines.

STOPS+ intervention

The STOPS+ intervention will consist of the ETAU plus the following components;

1. Supervision by a trained family member for dispensing and administering medication

A family member who is living with the patient for a minimum of 6 months prior to recruitment to the trial will be nominated by the patient. The family member will dispense medication, observe that is has been taken correctly and record this information on a simple sheet of paper designed and used in the previous STOPS trial.[8] Family members will be reminded to administer the medication by an automated text message system, which will also request a response once the medication has been taken correctly. The text message reminders will be sent every day for the first three months after starting the treatment. If the family member does not have access to a mobile phone, a handset and training in using it will be provided. A Multi-Purpose technician (MPT) from each PHC centre will provide training to family members for the storage and administration of the medication as well as recording when the medication has been taken correctly.

2. Treatment for schizophrenia at PHC level supervised by mental health professionals

Ongoing management and monitoring of the patient's schizophrenia will take place at their PHC centre by the PHC physician, under the supervision of the treating psychiatrist with the help of the mobile technology platform SMART (Smartphone for Monitoring, Assessment and Remote Treatment). SMART will provide two-way communication between mental health care professionals. One month's supply of medication will be made available, via their PHC, to the family member. The automated messaging system will also remind the family member about collecting the following month's supply.

3. Monitoring the availability of essential psychotropic medication and their side effects

A kit to monitor the supply of medication will be used to monitor the availability and supply of psychotropic medication. This will consist of a) a cupboard dedicated specifically for the storage of psychotropic medication, b) a tracking sheet used to monitor the stock levels c) a simple checklist for monitoring side-effects of antipsychotics.

Assessment of barriers and facilitators of implementation of the intervention

For peer review only

The use of theory to inform the development of behaviour change interventions is strongly advocated by experts in the field. We have designed the process of implementation using theory developed for implementation and tested previously in UK Primary Care Practice [14,15]. We will evaluate the barriers and facilitators to implementation of the intervention using qualitative methods. We will hold at least four focus groups (with patients and carers, multipurpose technicians, traditional and faith healers and primary care clinicians) in which we will explore the barrier and facilitators to supervising the treatment of schizophrenia in the community, modification of the STOPS programme to STOPS+ for use in the community and the present status of schizophrenia care in the community

Patient and Public Involvement

STOPS+ is underpinned by culturally appropriate and context-bespoke patient and public community engagement and involvement at every stage of the project (intervention development, RCT and process evaluation). The STOPS+ intervention will be refined with input from the key stakeholder groups including patients, carers, health professionals, local councillors, community leaders such as religious leaders and community experts and policy makers. Small group meetings using local Jirgas (Jirga is traditional, local participatory and decision-making body which is well-established in Pathan culture and brings together local tribal, ethnic and religious leaders) will help in community engagement.

OUTCOMES AND MEASURES

Primary outcome measure

In view of the pragmatic nature of the trial, we will use two distinct primary outcomes:
(i) The primary clinical outcome will be the level of functioning, measured using the Global Assessment of Functioning (GAF) scale.[16] GAF is a commonly used tool in schizophrenia, particularly for the pragmatic trials.[17]
(ii) The primary process outcome will be adherence to treatment regimen. The adherence to treatment will be measured using a questionnaire adapted from Herz et al (2000).[18] This measure for assessing treatment adherence was successfully used in our previous STOPS study.[8]

Secondary outcomes:

The details of secondary outcomes and the standardised instruments for measuring these are given in Table 1. These instruments will measure secondary outcomes such as physical health outcomes, family/caregivers burden and internalised stigma.

TABLE 1 HERE PLEASE

Eligibility criteria

Patients will be eligible for inclusion if they;

a) have a diagnosis of schizophrenia or schizoaffective disorder based on the International Classification of Disease 10 (ICD-10) criteria assessed using the Mini-International Neuropsychiatric Interview [19]
b) are aged between 17-65 years
c) do not meet the criteria for remission as defined by the Remission in Schizophrenia Working Group [30]
d) have capacity and are able to give informed consent

Exclusion criteria will be kept to a minimum to ensure that the study can examine the real world implementation of the STOPS+. The patients who can't participate in the study due to one of following reason will be excluded I) a serious or unstable medical illness, II) evidence of learning disability, III) severe drug dependence requiring treatment and/or detoxification IV) pregnant or breast-feeding.

Duration of treatment and follow-up:

Both groups will be followed up for 12 months after recruitment in the study. The follow up using face-to-face assessments will be carried out by trained research assistants in a health facility which is not part of participating PHCs to preserve the blindness of clusters. All of the outcomes will be measured at baseline, at 6 months and at 12 months by research assistants.

Participant Identification

Participants will be identified from two possible sources i) patients living in the area of the participating PHCs and presenting at psychiatry outpatients services of the hospitals in the area (ii) potentially eligible patients identified by the primary health care workers during community engagement or attending the PHCs. Eligible participants will be informed about the trial orally by the PHC staff and written information will be provided in the local language (Urdu). Those interested in taking part will be asked to give written informed consent. For participants who cannot read and write, witnessed oral consent and a thumb print in lieu of a signature will be used. The witness will not be a member of the research team. The procedure has been used in our previous trials in the area.[31]

Randomisation and allocation concealment

Randomisation will occur at the PHC level, where PHCs will be allocated at a ratio of 1:1 to either deliver the STOPS+ intervention or ETAU. Twenty-four PHCs will be recruited to the study, 12 will be randomised to deliver ETAU and 12 will be randomised to deliver the STOPS+ programme. PHC randomisation will be stratified by urban/rural setting. Randomisation will be carried out by an independent statistician at the Khyber Medical University (KMU), Peshawar by using a remote computer generated random sequence. Randomisation and will be based on the list of primary care centres in Peshawar, provided by the health department of KP. The urban/rural classification is determined as per the Peshawar district council. It is not possible to blind study participants from their treatment arm allocation. However, the assessment team that conducts the baseline and follow up assessments (6 and 12 months) will be masked to the treatment arm of the cluster. The outcome measures will be administered by trained research assessment teams (outcome

For peer review only

assessors), who are independent of the intervention team and the team involved in consenting and screening. The outcome assessment will not be carried out at any participating PHC. The assessment teams will be told that they are evaluating two interventions and that there is genuine equipoise about which one is better. Study participants will be instructed to not discuss the PHC they are attending or the treatment details. During all assessments, the primary outcome measures (treatment adherence scale and GAF) will be completed first to minimise the risk of bias in the event of unmasking and, if it occurs, the point of unmasking will be recorded. Sensitivity analyses will be carried out to assess the effect of unmasking on the primary outcomes. The data linking each PHC with treatment allocation status is kept separate from the outcome dataset until the time of the final analysis. The trial statistician will be blind to allocation status.

Screening, Data collection and follow up

After identification of potentially eligible patients, they will first be orally informed about the trial by one of the health workers in the local Pushto language (MT in the community or a health professional in one of the health facilities mentioned above). They will be asked whether they agree that a member of the research team will provide them with further information about the research. If permission is given, a research assistant will meet with the patient and will request informed consent. The informed consent for participation in the study will be taken within one week of the first contact. Participants will be free to decline to participate or withdraw at any time without their routine health care being affected. Before taking part in any interviews, oral and written information about the study and its purpose will be provided to respondents in the local language.

After obtaining informed consent the research assistant will decide whether the patient meets the inclusion criteria and have a family member who is willing to supervise the treatment as specified in the STOPS+ procedures. If the patient is not eligible from the screening assessment the reason for ineligibility will be explained to the patient and they will be signposted for further advice and to continue to receive treatment at their routine health facility. Eligible patients will be provided with a further participant information sheet about the trial. Additionally, for those in the intervention arm, the nominated relative will receive a participant information sheet about the trial and about the tasks required in supervising the treatment If nominated family member is available with the patient, this information will be provided at the same time. If the family member is not available, patients and the family member will be asked to visit the health facility again within one week. The patient and the relative will each be given the opportunity to ask any questions about the trial. The information sheets are provided in Urdu. For illiterate patients the research assistants will explain the same information in local Pushto language or in Urdu. Following the informed consent, the baseline data will be collected.

Sample size calculation

A mean difference of 6 on the primary clinical outcome measure (GAF) with a pooled study standard deviation of 15 was observed in our previous trial in the same setting.[11] A recent indicated minimum clinically important difference for the GAF in schizophrenia patients has been given as a mean difference of 4).[32] A total sample size of 526 i.e. 263 patients in each arm (recruited across 24 PHCs, with an average cluster size of 22 patients) has over

For peer review only

90% power to detect a mean difference of 6 and 85-90% power to detect a more modest mean difference of 4 in the GAF given a two-tailed significance level of 0.05 and based on the following parameter assumptions: i) baseline-outcome correlation of 0.5, repeated measures correlation of 0.7 [33] and loss to follow up of 20% (patient-level parameters); ii) intracluster correlation coefficient of 0.01 [34] and coefficient of variation in cluster sizes of 0.65 (PHC-level parameters).[35] The primary endpoint for the GAF is the ‘average’ GAF across 6 and 12 months follow up.

This sample size has 90% power to detect at least a 20% absolute difference in the treatment adherence rate between the intervention arm compared to the ETAU arm at 6 and/or 12 months follow up based on two-tailed alpha of 0.025 (to account for multiple testing in respect of 6 and 12 month testing). This is based on an observed ≈20% higher level of adherence within the intervention compared to ETAU arms in the original STOPS trial at 3 and 12 months follow up,[11] and further assuming the design effect PHC-level parameters as noted in (ii) above. Thus, the study is adequately powered to detect significant differences (at the level of 2-tailed alpha of 0.05 in each case) across the two distinct primary outcomes: i) the primary clinical outcome (GAF) and ii) the primary process outcome (medication adherence).

Trial management and monitoring

The STOPS+ trial will be monitored in line with the protocol and the trial standard operating procedures. An independent Trial Steering Committee (TSC) which will have service user representatives will monitor trial progress. The TSC is empowered to independently review the ethical and data management procedures. A Data and Safety Monitoring Board (DSMB) will be convened to ensure the safety of participants and the integrity of the data. The PHCs in the trial will receive training around safety reporting and additionally, participants and their family members will be informed of how to report concerns and safety issues, whilst they are participating in the trial. The Project Management Committee (PMC) will be responsible for setting up the trial, ongoing management and monitoring, promotion of the study, training and the interpretation of the results.

Safety and Adverse events reporting

All adverse events and serious adverse events (SAEs) that are reported by the participant, family member or identified by the trial team or PHC staff members during the trial will be recorded and reported following trial safety reporting procedures. Related and unexpected SAEs will be reported to the local Research Ethics Committee (REC), DSMB and TSC within 15 days. The DSMB will review all safety data and make any decisions regarding early termination of the study in line with their respective terms of reference.

Data management

All data will be managed in line with the protocol and will be stored securely following the KMU and Keele University standard policy on data storage and confidentiality. Individual participant data will be pseudonymised through the use of a unique study ID and will be stored securely and separately from any identifiable data. Access to data will be restricted to members of the research team. Outcome data will be entered into an electronic database

For peer review only

which will be stored on an encrypted secure network at KMU requiring a password to access. Data entry will be quality checked and coded using standard processes.

Statistical analysis

Statistical analysis will be performed blind to cluster allocation. Primary analysis will be on an intention-to-treat (ITT) basis, with all participants analysed in their cluster. Between-group differences for both primary outcome measures (GAF and medication adherence) will be analysed using longitudinal mixed models that accommodate clustering at the (upper/hierarchical) practice level, as well as repeated outcome measurements across 3 time points (baseline, 6 and 12 months) per participant. i.e. linear/logistic mixed models for numerical and categorical outcomes, respectively, including PHC and participant as random factors. These models will be used to quantify the absolute between group difference in the primary clinical outcome (GAF) and odds ratio (and extracted absolute difference) for the primary process outcome (% adherence) – using linear and logistic models, respectively. For the GAF, the primary endpoint is the 'average' GAF over 6 and 12 months using available and modelled data to estimate the combined summary average mean difference in GAF between treatment arms over the 6 and 12 months timeline; statistical significance in this case being given against two-tailed p=0.05. Though the 'average' is the single primary endpoint for the GAF, we will also evaluate the treatment-by-time interaction to give estimates of between-arm mean differences at the individual 6 and 12 months follow-up time points (secondary endpoints).

For medication adherence, between-group comparison at both 6 and 12 months are primary (derived through treatment-by-time interaction within the generalised mixed model) – hence, the requirement to take into account the multiple-testing by assessing significance against a more conservative two-tailed p=0.025. Estimates of between-group mean differences will be given including 95% confidence intervals and p-values. The mixed models (detailed above) will be adjusted for age, gender, baseline GAF score and corresponding baseline score of the outcome being measured as individual-patient level covariates, PHC-level characteristics (PHC size), stratification variable (rural/urban) and a random effect for the PHC. Mixed model analysis fulfils the ITT principle with missing data being accounted for under the missing at random (MAR) assumption.

Cost effectiveness

The cost of STOPS+ will be evaluated against ETAU using the Service Receipt Inventory (SRI),[36] which has been used in previous trials in Pakistan.[19] This will include the cost of primary and outpatient care visits, inpatient admissions, drug regimens, diagnostic tests, travel time, transport costs and lost days from work. Costs will be subsequently linked to outcomes in order to establish whether STOPS+ represents a cost-effective use of resources (e.g. whether the additional cost of supervision by the relative are offset by the reduced hospital care and/or improvements in the clinical outcome – GAF and QALY). Cost-effectiveness planes and acceptability curves will evaluate the uncertainty in estimates as well as the probability of STOPS+ being cost-effective across a range of willingness-to-pay thresholds. Adjusted analysis will be carried to accommodate clustering of PHCs.

Ethics and dissemination

For peer review only

Ethical approval to conduct this trial have been obtained from Keele University Ethical review Panel (reference: MH-190017) and Khyber Medical University Ethical Review Board (ref: DIR-KMU-EB/ST/000648). Results will be disseminated through peer reviewed journals and academic conferences. The trial is being conducted in accordance with the International Conference on Harmonisation/Good Clinical Practice (ICHGCP) Guidelines (1996) which are consistent with principles of Declaration of Helsinki (1996) Ethical Principles for Medical Research Involving Human Subjects.

Process evaluation

Patients and relatives, primary care physicians and multipurpose technician from participating PHCs in both arms will be invited to take part in a semi-structured interview to assess the acceptability of intervention and its implementation in the health system.[37] These interviews will last approximately 30-40 minutes and will be audio recorded with permission. A minimum of 20 individual semi-structured interviews [38] will be conducted (continuing until data saturation is reached)[39,40] with patients and their relatives to explore a) the acceptability of the STOPS+ intervention; b) the experience/burden of the training and support for the patient's family member; c) barriers and facilitators to medication adherence and overall satisfaction with the intervention and trial participation. A minimum of 10 interviews will be conducted with PHC physicians. In addition, we will conduct interviews with at least 5 decision-makers with responsibilities for developing or implementing health policy in the directorate of health in order to capture their perceptions about the feasibility, the benefits, challenges and acceptability of STOPS+ in the health system. Participants for these interviews will be purposively sampled to stratify by gender and urban/rural setting. A semi-structured topic guide will be used to facilitate interviews. Informed consent will be obtained prior to the start of the interview and will include consent for the use of direct pseudonymised quotes.

An inductive, exploratory framework will be adopted using thematic analysis based on the principles of grounded theory.[41,42] A sample of early transcripts will be independently coded by the research team and a coding framework agreed upon. This framework will then be applied to subsequent coding. Coded data will be analysed independently by qualitative study leads at Keele and KMU to develop categories and themes to be discussed in wider research team meetings. Constant comparison will be used to explore connections within and across transcripts and codes, highlighting consistencies and variation.[43] Analysis will follow an iterative process, with emergent findings used to further refine topic guides for subsequent interviews.

DISCUSSION

People suffering from schizophrenia represent one of the most disadvantaged groups in low- and middle-income countries facing a wide mental health treatment gap. A number of psychosocial interventions have been evaluated in RCTs and there is consensus that the treatment of schizophrenia should combine anti-psychotic medication and psychosocial interventions.[44] However there is limited evidence how these interventions can be implemented in real world settings.

For peer review only

The proposed study adopts an implementation approach addressing systemic barriers to reduce treatment gap for schizophrenia in the community. The STOPS+ intervention aims to address three major implementation barriers (i) improving supply of antipsychotic medication at primary care level (ii) involving primary care workers in the treatment of schizophrenia (iii) improving treatment adherence by involving family members in supervising treatment. The STOPS+ approach therefore goes beyond most psychosocial interventions evaluated in treatment of schizophrenia in LMIC countries [45, 46] in addressing the health system, community and service users related factors to improve access to treatment.
A unique aspect of the trial is, that it is powered for process and clinical outcomes. These two primary outcomes are: (i) a clinical outcome measure – Global Assessment of Functioning and (ii) a process outcome measure – adherence to medication. This combined with a process evaluation carried out after completion of the cluster RCT will provide much needed evidence on the implementation of community-based interventions for schizophrenia in LMIC countries settings. The WHO Mental Health Action Plan (2013-2020) requires that there is an absolute increase of 20% in service coverage for severe mental disorders.[46] The results of the trial may help to understand the processes and implementation strategies to achieve that goal and could provide an effective public health intervention for schizophrenia.[47]

A major strength of the STOPS intervention is that it is based on an infectious disease model that has been successfully used in improving treatment adherence for Tuberculosis i.e. DOTS (http://www.searo.who.int/tb/topics/what_dots/en/) as a public health intervention. The DOTS (Directly Observed Treatment Short course) model is currently being used in most LMIC countries, including the settings of this trial. This can potentially help in integration of the care for a non-communicable chronic disorder like schizophrenia in the public health systems of LMIC countries.

Competing interests statement: SF in the past 3 years has received honoraria and speaker's fees from Otsuka, Lundbeck and Sunovian pharma and also research funding from Sunovian Pharmaceutical. MMuH and MFK received funding from different pharmaceuticals to attend scientific meetings.

Acknowledgements: Krysia Dziedzic is an NIHR Senior Investigator. The views expressed in this paper are those of the authors and not necessarily those of the NHS, NIHR, or the Department of Health and Social Care.

For peer review only

References

1 GBD 2017 Disease and Injury Incidence and Prevalence Collaborators. Global, regional, and national incidence, prevalence, and years lived with disability for 354 diseases and injuries for 195 countries and territories, 1990–2017: a systematic analysis for the Global Burden of Disease Study 2017*. The Lancet. 2018;**392**(10159)*:1789-1858

2 Charlson FJ, Ferrari AJ, Santomauro DJ, et al. Global Epidemiology and Burden of Schizophrenia: Findings From the Global Burden of Disease Study 2016. *Schizophrenia Bulletin* 2018;**44**(6):1195-1203.

3 Lora A, Kohn R, Levav I, et al. Service availability and utilization and treatment gap for schizophrenic disorders: a survey in 50 low- and middle-income countries. *Bull World Health Organ* 2012;**90**(1):47-54B.

4 Lacro JP, Dunn LB, Dolder CR, et al. Prevalence of and risk factors for medication nonadherence in patients with schizophrenia: a comprehensive review of recent literature. J Clin Psychiatry 2002;63:892–909.

5 Irfan M. Integration of mental health in primary care in Pakistan. *J Postgrad Med Inst* 2013; 27(4):349-51.

6 Large M, Farooq S, Nielssen O, et al. Relationship between gross domestic product and duration of untreated psychosis in low- and middle-income countries. *Br J Psychiatry* 2008;**193**(4):272-278.

7 Farooq S, Burns J, Sumathipala A, et al. A Global Mental Health Fund for the treatment of Serious Mental Illness in Low Income and Middle Income Countries. *The Lancet Psychiatry 2016;***3**(6):182-183.

8 Weiden PJ and Olfson M. Cost of relapse in schizophrenia. *Schizophr Bull* 1995;**21**(3):419-429.

9 Farooq S, Large M, Nielssen O, et al. The relationship between the duration of untreated psychosis and outcome in low-and-middle income countries: a systematic review and meta analysis. *Schizophr Res* 2009;**109**(1-3):15-23.

10 McDaid D and Park A-L. Counting all the costs: the economic impact of comorbidity. *Karger Publishers* 2014.

11 Farooq S, Nazar Z, Irfan M, et al. Schizophrenia medication adherence in a resource-poor setting: randomised controlled trial of supervised treatment in out-patients for schizophrenia (STOPS). *Br J Psychiatry* 2011;**199**(6):467-472.

12 Campbell M, Fitzpatrick R, Haines A, et al. Framework for design and evaluation of complex interventions to improve health. *BMJ* 2000;**321**(7262):694-696.

For peer review only

13 Government of Khyber Pakhtunkhwa. Mental Health And Psychosocial Support: Strategy And 5-Year Multi-Sectoral Action Plan For Khyber Pakhtunkhwa (2017-2022). *Government of Khyber Pakhtunkhwa* 2017.

14 Grol R, Wensing M, Eccles M: Improving Patient Care - The Implementation of Change in Clinical Practice. 2004, London: Elsevier

15 Michie S, van Stralen MM, West R: The behaviour change wheel: a new method for characterising and designing behaviour change interventions. Implement Sci. 2011, 6: 42-10.1186/1748-5908-6-42.

16 American Psychiatric Association. DSM-IV-TR: Diagnostic and statistical manual of mental disorders, text revision. Washington, DC: American Psychiatric Association 2000;**75**.

17 Crawford MJ, Killaspy H, Barnes TRE, et al. Group art therapy as an adjunctive treatment for people with schizophrenia: multicentre pragmatic randomised trial. *BMJ* 2012;344:e846

18 Herz MI, Lamberti S, Mintz J, et al. A program for relapse prevention in schizophrenia. A controlled study. *Arch Gen Psychiatry* 2000;57:277–283.

19 Sheehan DV, Lecrubier Y, Sheehan KH, et al. The Mini-International Neuropsychiatric Interview (M.I.N.I.): the development and validation of a structured diagnostic psychiatric interview for DSM-IV and ICD-10. *J Clin Psychiatry* 1998;**59**(Suppl 20):22-33.

20 Overall, JE, & Gorham, DR. The Brief Psychiatric Rating Scale. *Psychological Reports* 1962;10(3):799–812.

21 Pai S & Kapur RL. The burden on the family of a psychiatric patient: development of an interview schedule. *Br J Psychiatry* 1981;**138**:332-335.

22 Lysaker PH, Davis LW, Warma DM, Strasburger A & Beattie N. Stigma, social function and symptoms in schizophrenia and schizoaffective disorder: associations across 6 months**.** *Psychiatry Research* 2007;14:89-95.

23 Waddell L, Taylor M. A new self-rating scale for detecting atypical or second-generation antipsychotic side effects. *Journal Psychopharmacology* 2008; 22:238

24 Chisholm D, Knapp MR, Knudsen HC, Amaddeo F, Gaite L, van Wijngaarden B. Client Socio-Demographic and Service Receipt Inventory--European Version: development of an instrument for international research. EPSILON Study 5. European Psychiatric Services: Inputs Linked to Outcome Domains and Needs.
Br J Psychiatry Suppl. 2000;(39):28-33.

25 The EuroQol Group (1990). EuroQol-a new facility for the measurement of health-related quality of life. *Health Policy* 1990;16(3):199-208.

26 Guy W (ed). ECDEU Assessment Manual for Psychopharmacology. Rockville, MD: US Department of Heath,Education, and Welfare Public Health Service Alcohol, Drug Abuse, and Mental Health Administration, 1976 Busner J, Targum SD. The clinical global

For peer review only

impressions scale: applying a research tool in clinical practice. *Psychiatry (Edgmont)* 2007;4(7):28-37

27 Skinner HA. The Drug Abuse Screening Test. *Addictive Behavior* 1982;*7*(4): 363–371.

28 Kroenke K, Spintzer RL, Williams JB. The PHQ-9: validity of a brief depression severity measure. Journal of General Internal Medicine 2001;16(9):606-613

29 Osman A, Bagge CL, Gutierrez PM, Konick LC, Kopper BA, Barrios FX., The Suicide Behaviours Questionnaire-Revised (SBQ-R): Validation with clinical and nonclinical samples. *Assessment*. 2001 (5):443-54

20 Andreasen NC, Carpenter WT Jr, Kane JM, et al. Remission in schizophrenia: proposed criteria and rationale for consensus. *Am J Psychiatry* 2005;**162**(3):441-449.

31 Rahman A, Hamdani SU, Awan NR, et al. (2016). Effect of a Multicomponent Behavioural Intervention in Adults Impaired by Psychological Distress in a Conflict-Affected Area of Pakistan: A Randomized Clinical Trial. *JAMA* 2016;316(24):2609-2617.

32 Amri I, Millier A, Toumi M. PMH2 - Minimum Clinically Important Difference in the Global Assessment Functioning in Patients with Schizophrenia. *Value in Health* 2014;17:A765–A766.

33 Vickers AJ. How many repeated measures in repeated measures designs? Statistical issues for comparative trials. *BMC Med Res Methodol* 2003;**3**:22.

34 Adams G, Gulliford MC, Ukoumunne OC, et al. Patterns of intra-cluster correlation from primary care research to inform study design and analysis. *J Clin Epidemiol* 2004;**57**(8):785-94.

35 Eldridge SM, Ashby D and Kerry S. Sample size for cluster randomized trials: effect of coefficient of variation of cluster size and analysis method. *Int J Epidemiol* 2006;35(5):1292-300.

36 Chisholm D, Knapp MR, Knudsen HC, et al. Client socio-demographic and service receipt inventory--european version: development of an instrument for international research. EPSILON study 5. European psychiatric services: inputs linked to outcome domains and needs. *Br J Psychiatry Suppl* 2000;39:s28–33.

37 O'Cathain A. A practical guide to using qualitative research with randomized controlled trials. Oxford University Press 2018.

38 Malterud K, Siersma VD and Guassora AD. Sample size in qualitative interview studies: guided by information power. *Qualitative health research* 2016;26(13):1753-1760.

39 Saunders B, Sim J, Kingstone T, et al 2018. Saturation in qualitative research: exploring its conceptualization and operationalization. *Quality & quantity* 2018;52(4):1893-1907.

For peer review only

40 Sim J, Saunders B, Waterfield J, et al. Can sample size in qualitative research be determined a priori?. *International Journal of Social Research Methodology 2018;*21(5):619-634.

41 Glaser BG and Strauss AL. Discovery of grounded theory: Strategies for qualitative research. Routledge 2017.

42 Braun V, Clarke V, Hayfield N, et al. Thematic analysis. Handbook of research methods in health social sciences. Singapore: Springer 2018:1-18.

43 Nowell LS, Norris JM, White DE, et al. Thematic analysis: Striving to meet the trustworthiness criteria. *International Journal of Qualitative Methods* 2017;16(1): p.1609406917733847.

44 Asher L, Patel V, De Sliva MJ. Community-based psychosocial interventions for people with schizophrenia in low and middle-income countries: systematic review and meta-analysis. *BMC Psychiatry* 2017;**17**:355.

45 Xu DR, Xiao S, He H, et al. Lay health supporters aided by mobile text messaging to improve adherence, symptoms, and functioning among people with schizophrenia in a resource-poor community in rural China (LEAN): A randomized controlled trial. *PLoS Med* 2019;**16**(4):e1002785.

46 Kaddick H, Horney B, McKenzie J, Tilley H. Investing in mental health in low and middle income countries. Overseas Development Institute, London 2016.

47 Farooq, S. Public health and physician focused strategies to improve medication adherence in psychotic disorders. *World Psychiatry* 2013;12(3):238-239.

Authors' contributions: TAS wrote first draft. All authors contributed to the conceptualisation and the design of the study. SF obtained the funding for the study, conceived and developed the intervention and study design, and led creation of the team. MFK, ZuH, MMuH, AA and US secured the study sites and contributed to design and development of intervention, community engagement and qualitative studies leading to modification of intervention. LD contributed to design and led qualitative studies for intervention development and process evaluation. US, TAS, ZuH and MER contributed to writing ethics applications and obtaining the ethics approvals. ML contributed significantly to the study design, analysis plan and economic evaluation part of the protocol. AR,LD, KD, CDM provided critical review and contributed to the design of the study. All authors read and revised the initial manuscript and approved the final version.

Funding statement – This work is supported by the Medical Research Council UK, as part of their Global Alliance for Chronic Disease programme grant number MR/S00243X/1.

Table 1. Description of outcome measures and instruments

	Domains measured	Measure
	Sociodemographics	Age<br>Gender<br>Years in education<br>Marital status<br>Work status<br>Mini-Neuropsychiatric Interview (MINI) [19]
	Physical health	Body Mass Index (BMI)<br>Blood Pressure<br>Waist Circumference
**Primary outcomes**		
	Level of functioning	Global Assessment of Functioning (GAF) [16]
	Level of adherence	Treatment adherence rating scale [18]
**Secondary outcomes**		
	Mental state and psychiatric symptoms	Brief Psychiatric Rating Scale [20]
	Caregivers' burden	Family Burden Scale [21]
	Perceived stigma	Internalised Stigma of Mental Illness [22]
	Side effects of antipsychotic medication	Glasgow Antipsychotic Side-Effects Scale [23]
	Cost of care	Client Service Receipt Inventory [24]
	Quality of life and cost effectiveness	EuroQol (EQ5D) [25]
	Illness severity, improvement and response to treatment	Clinical Global Impression Scale [26]
	Drug use	DAST Drug Screening Tool [27]
	Depression	Patient Health Questionnaire [28]
	Suicide Ideation	Suicide Behaviours Questionnaire Revised [29]

For peer review only

For peer review only

The editor

BMJ

Re:

We are pleased to submit the protocol article for the above mentioned study. The article describes methodology to evaluate an innovative approach for reducing treatment gap for schizophrenia.

The article is 4690 words in length. This is due to fact that we had to describe intervention, settings and methods in sufficient details. We feel that it is important to provide sufficient details about the intervention and methods for the reviewers and readers to allow the replication and clear understanding of the study. I hope you will agree with this and accept the article. We will of course be happy to respond to reviewers' comments or the suggestions from editorial team.

With best wishes

Professor Saeed Farooq

For peer review only

SPIRIT
STANDARD PROTOCOL ITEMS: RECOMMENDATIONS FOR INTERVENTIONAL TRIALS

SPIRIT 2013 Checklist: Recommended items to address in a clinical trial protocol and related documents*

Section/item	Item No	Description	Addressed on page number
**Administrative information**			
Title	1	Descriptive title identifying the study design, population, interventions, and, if applicable, trial acronym	______8_______
Trial registration	2a	Trial identifier and registry name. If not yet registered, name of intended registry	______39_______
	2b	All items from the World Health Organization Trial Registration Data Set	_______*______
Protocol version	3	Date and version identifier	_______1______
Funding	4	Sources and types of financial, material, and other support	_______5______
Roles and responsibilities	5a	Names, affiliations, and roles of protocol contributors	_______5,6,7____
	5b	Name and contact information for the trial sponsor	_______5*______
	5c	Role of study sponsor and funders, if any, in study design; collection, management, analysis, and interpretation of data; writing of the report; and the decision to submit the report for publication, including whether they will have ultimate authority over any of these activities	______na_______
	5d	Composition, roles, and responsibilities of the coordinating centre, steering committee, endpoint adjudication committee, data management team, and other individuals or groups overseeing the trial, if applicable (see Item 21a for data monitoring committee)	______na_______

For peer review only - http://bmjopen.bmj.com/site/about/guidelines.xhtml

Introduction

Background and rationale	6a	Description of research question and justification for undertaking the trial, including summary of relevant studies (published and unpublished) examining benefits and harms for each intervention	_______10,11____
	6b	Explanation for choice of comparators	________11______
Objectives	7	Specific objectives or hypotheses	_______12_______
Trial design	8	Description of trial design including type of trial (eg, parallel group, crossover, factorial, single group), allocation ratio, and framework (eg, superiority, equivalence, noninferiority, exploratory)	________?______

Methods: Participants, interventions, and outcomes

Study setting	9	Description of study settings (eg, community clinic, academic hospital) and list of countries where data will be collected. Reference to where list of study sites can be obtained	_______17,18____
Eligibility criteria	10	Inclusion and exclusion criteria for participants. If applicable, eligibility criteria for study centres and individuals who will perform the interventions (eg, surgeons, psychotherapists)	_______18_______
Interventions	11a	Interventions for each group with sufficient detail to allow replication, including how and when they will be administered	_____14,15,16,17_
	11b	Criteria for discontinuing or modifying allocated interventions for a given trial participant (eg, drug dose change in response to harms, participant request, or improving/worsening disease)	______26_______
	11c	Strategies to improve adherence to intervention protocols, and any procedures for monitoring adherence (eg, drug tablet return, laboratory tests)	_______16, 34____
	11d	Relevant concomitant care and interventions that are permitted or prohibited during the trial	_______26_______
Outcomes	12	Primary, secondary, and other outcomes, including the specific measurement variable (eg, systolic blood pressure), analysis metric (eg, change from baseline, final value, time to event), method of aggregation (eg, median, proportion), and time point for each outcome. Explanation of the clinical relevance of chosen efficacy and harm outcomes is strongly recommended	12,13,21,22,23,24
Participant timeline	13	Time schedule of enrolment, interventions (including any run-ins and washouts), assessments, and visits for participants. A schematic diagram is highly recommended (see Figure)	_______9_______

For peer review only - http://bmjopen.bmj.com/site/about/guidelines.xhtml

Sample size	14	Estimated number of participants needed to achieve study objectives and how it was determined, including clinical and statistical assumptions supporting any sample size calculations	___26,27_______
Recruitment	15	Strategies for achieving adequate participant enrolment to reach target sample size	______27_______
**Methods: Assignment of interventions (for controlled trials)**			
Allocation:			
Sequence generation	16a	Method of generating the allocation sequence (eg, computer-generated random numbers), and list of any factors for stratification. To reduce predictability of a random sequence, details of any planned restriction (eg, blocking) should be provided in a separate document that is unavailable to those who enrol participants or assign interventions	______19_______
Allocation concealment mechanism	16b	Mechanism of implementing the allocation sequence (eg, central telephone; sequentially numbered, opaque, sealed envelopes), describing any steps to conceal the sequence until interventions are assigned	_______8______
Implementation	16c	Who will generate the allocation sequence, who will enrol participants, and who will assign participants to interventions	_______19______
Blinding (masking)	17a	Who will be blinded after assignment to interventions (eg, trial participants, care providers, outcome assessors, data analysts), and how	____19,20______
	17b	If blinded, circumstances under which unblinding is permissible, and procedure for revealing a participant's allocated intervention during the trial	_____________
**Methods: Data collection, management, and analysis**			
Data collection methods	18a	Plans for assessment and collection of outcome, baseline, and other trial data, including any related processes to promote data quality (eg, duplicate measurements, training of assessors) and a description of study instruments (eg, questionnaires, laboratory tests) along with their reliability and validity, if known. Reference to where data collection forms can be found, if not in the protocol	__21,22,23,24___
	18b	Plans to promote participant retention and complete follow-up, including list of any outcome data to be collected for participants who discontinue or deviate from intervention protocols	______na_______

Data management	19	Plans for data entry, coding, security, and storage, including any related processes to promote data quality (eg, double data entry; range checks for data values). Reference to where details of data management procedures can be found, if not in the protocol	___32,33,34_____
Statistical methods	20a	Statistical methods for analysing primary and secondary outcomes. Reference to where other details of the statistical analysis plan can be found, if not in the protocol	______28,29____
	20b	Methods for any additional analyses (eg, subgroup and adjusted analyses)	_______29,30___
	20c	Definition of analysis population relating to protocol non-adherence (eg, as randomised analysis), and any statistical methods to handle missing data (eg, multiple imputation)	______29_______
**Methods: Monitoring**			
Data monitoring	21a	Composition of data monitoring committee (DMC); summary of its role and reporting structure; statement of whether it is independent from the sponsor and competing interests; and reference to where further details about its charter can be found, if not in the protocol. Alternatively, an explanation of why a DMC is not needed	______34_______
	21b	Description of any interim analyses and stopping guidelines, including who will have access to these interim results and make the final decision to terminate the trial	______30_______
Harms	22	Plans for collecting, assessing, reporting, and managing solicited and spontaneously reported adverse events and other unintended effects of trial interventions or trial conduct	___35,36,45_____
Auditing	23	Frequency and procedures for auditing trial conduct, if any, and whether the process will be independent from investigators and the sponsor	___33,34,35,36__
**Ethics and dissemination**			
Research ethics approval	24	Plans for seeking research ethics committee/institutional review board (REC/IRB) approval	______39_______
Protocol amendments	25	Plans for communicating important protocol modifications (eg, changes to eligibility criteria, outcomes, analyses) to relevant parties (eg, investigators, REC/IRBs, trial participants, trial registries, journals, regulators)	______na_______

For peer review only - http://bmjopen.bmj.com/site/about/guidelines.xhtml

Consent or assent	26a	Who will obtain informed consent or assent from potential trial participants or authorised surrogates, and how (see Item 32)	_______20________
	26b	Additional consent provisions for collection and use of participant data and biological specimens in ancillary studies, if applicable	_______na________
Confidentiality	27	How personal information about potential and enrolled participants will be collected, shared, and maintained in order to protect confidentiality before, during, and after the trial	____31,32,33____
Declaration of interests	28	Financial and other competing interests for principal investigators for the overall trial and each study site	_______39________
Access to data	29	Statement of who will have access to the final trial dataset, and disclosure of contractual agreements that limit such access for investigators	_______40________
Ancillary and post-trial care	30	Provisions, if any, for ancillary and post-trial care, and for compensation to those who suffer harm from trial participation	________39______
Dissemination policy	31a	Plans for investigators and sponsor to communicate trial results to participants, healthcare professionals, the public, and other relevant groups (eg, via publication, reporting in results databases, or other data sharing arrangements), including any publication restrictions	____40,41______
	31b	Authorship eligibility guidelines and any intended use of professional writers	________*______
	31c	Plans, if any, for granting public access to the full protocol, participant-level dataset, and statistical code	________*______
**Appendices**			
Informed consent materials	32	Model consent form and other related documentation given to participants and authorised surrogates	_______*_______
Biological specimens	33	Plans for collection, laboratory evaluation, and storage of biological specimens for genetic or molecular analysis in the current trial and for future use in ancillary studies, if applicable	_______na________

*It is strongly recommended that this checklist be read in conjunction with the SPIRIT 2013 Explanation & Elaboration for important clarification on the items. Amendments to the protocol should be tracked and dated. The SPIRIT checklist is copyrighted by the SPIRIT Group under the Creative Commons "Attribution-NonCommercial-NoDerivs 3.0 Unported" license.

For peer review only - http://bmjopen.bmj.com/site/about/guidelines.xhtml

BMJ Open

Supervised Treatment in Outpatients for Schizophrenia Plus (STOPS+): Protocol for a cluster randomised trial of a community-based intervention to improve treatment adherence and reduce the treatment gap for schizophrenia in Pakistan

Journal:	*BMJ Open*
Manuscript ID	bmjopen-2019-034709.R1
Article Type:	Protocol
Date Submitted by the Author:	20-Mar-2020
Complete List of Authors:	Shepherd, Thomas; Keele University, School of Primary, Community and Social Care<br>Ul-Haq, Zia; Khyber Medical University, Institute of Public Health & Social Sciences<br>Ul-Haq, Mian; Lady Reading Hospital<br>Khan, Muhammad Firaz; Lady Reading Hospital<br>Afridi, Adil; Lady Reading Hospital<br>Dikomitis, Lisa; Keele University, School of Primary, Community and Social Care; Keele University, School of Medicine<br>Robinson, Michelle; Keele University, School of Primary, Community and Social Care<br>Lewis, Martyn; Keele University, School of Primary, Community and Social Care<br>Rahman, Atif; University of Liverpool, Child Mental Health Unit<br>Dziedzic, Krysia; Keele University, School of Primary, Community and Social Care<br>Saeed, Umaima; Khyber Medical University, Institute of Public Health & Social Sciences<br>Awan, Naila; Lady Reading Hospital<br>Mallen, Christian; Keele University, School of Primary, Community and Social Care; Midlands Partnership Foundation Trust<br>Farooq, Saeed; Keele University, School of Primary, Community and Social Care; Midlands Partnership Foundation Trust
**Primary Subject Heading**:	Global health
Secondary Subject Heading:	Mental health
Keywords:	MENTAL HEALTH, Clinical trials < THERAPEUTICS, PUBLIC HEALTH, PSYCHIATRY, Schizophrenia & psychotic disorders < PSYCHIATRY

For peer review only - http://bmjopen.bmj.com/site/about/guidelines.xhtml

I, the Submitting Author has the right to grant and does grant on behalf of all authors of the Work (as defined in the below author licence), an exclusive licence and/or a non-exclusive licence for contributions from authors who are: i) UK Crown employees; ii) where BMJ has agreed a CC-BY licence shall apply, and/or iii) in accordance with the terms applicable for US Federal Government officers or employees acting as part of their official duties; on a worldwide, perpetual, irrevocable, royalty-free basis to BMJ Publishing Group Ltd ("BMJ") its licensees and where the relevant Journal is co-owned by BMJ to the co-owners of the Journal, to publish the Work in this journal and any other BMJ products and to exploit all rights, as set out in our licence.

The Submitting Author accepts and understands that any supply made under these terms is made by BMJ to the Submitting Author unless you are acting as an employee on behalf of your employer or a postgraduate student of an affiliated institution which is paying any applicable article publishing charge ("APC") for Open Access articles. Where the Submitting Author wishes to make the Work available on an Open Access basis (and intends to pay the relevant APC), the terms of reuse of such Open Access shall be governed by a Creative Commons licence – details of these licences and which Creative Commons licence will apply to this Work are set out in our licence referred to above.

Other than as permitted in any relevant BMJ Author's Self Archiving Policies, I confirm this Work has not been accepted for publication elsewhere, is not being considered for publication elsewhere and does not duplicate material already published. I confirm all authors consent to publication of this Work and authorise the granting of this licence.

For peer review only

Supervised Treatment in Outpatients for Schizophrenia Plus (STOPS+): Protocol for a cluster randomised trial of a community-based intervention to improve treatment adherence and reduce the treatment gap for schizophrenia in Pakistan

Thomas A. Shepherd[1], Zia Ul Haq[2], Mian Muktar ul-Haq[3], Muhammad Firaz Khan[3], Adil Afridi[3], Lisa Dikomitis[1,6], Michelle E. Robinson[1], Martyn Lewis[1], Atif Rahman[4], Krysia Dziedzic[1], Umaima Saeed[2], Naila Riaz Awan[3] Christian D. Mallen[1, 5] & Saeed Farooq[1,5]

[1]School of Primary, Community and Social Care, Keele University, Staffordshire, ST5 5BG, UK

[2]Khyber Medical University, Phase V, Hayatabad, Peshawar, Khyber Pakhtunkhwa, 25100 Pakistan

[3]Lady Reading Hospital, Soekarno Rd, Pipal Mandi, Peshawar, Khyber Pakhtunkhwa 25000, Pakistan

[4]University of Liverpool, Foundation Building, Brownlow Hill, Liverpool, L69 7TX, UK

[5]Midlands Partnership Foundation Trust, Trust Headquarters, Trust Head Quarters Mellor House, Corporation St, Stafford ST16 3SR

[6] School of Medicine, Keele University Staffordshire, ST5 5BG, UK

Correspondence: Prof. Saeed Farooq, Faculty of Medicine and Health Sciences, Keele University, [1]School of Primary, Community and Social Care, Keele University, Staffordshire, ST5 5BG, UK

E mail: s.farooq@keele.ac.uk
Telephone: 01782 734973

Keywords: Psychosis, Low and middle Income Countries (LMIC), Drug Treatment, Directly Observed Therapy (DOTS)

Word Count: 5102

For peer review only - http://bmjopen.bmj.com/site/about/guidelines.xhtml

ABSTRACT

Introduction: There is a significant treatment gap, with only a few community-based services for people with schizophrenia in Low- and Middle-Income countries. Poor treatment adherence in schizophrenia is associated with poorer health outcomes, suicide attempts and death. We previously reported the effectiveness of STOPS (Supervised Treatment in Outpatients for Schizophrenia) for improving treatment adherence in patients with schizophrenia. However, STOPS was evaluated in a tertiary care setting with no primary care involvement, limiting its generalisability to the wider at-risk population. We aim to evaluate the effectiveness of STOPS+ in scaling up the primary care treatment of schizophrenia to a real-world setting.

Methods: The effectiveness of the STOPS+ intervention in improving the level of functioning and medication adherence in patients with schizophrenia in Pakistan will be evaluated using a cluster randomised controlled trial design. We aim to recruit 526 participants from 24 Primary Health Care Centres (PHCs) randomly allocated in 1:1 ratio to STOPS+ intervention and Enhanced Treatment as Usual (ETAU) arms. Participants will be followed-up for 12 months' post recruitment.

Analysis: The sample size is estimated for two outcomes (i) The primary clinical outcome is level of functioning, measured using the Global Assessment of Functioning (GAF) scale, and (ii) the primary process outcome is adherence to treatment regimen measured using a validated measure. An intention-to-treat approach will be used for the primary analysis.

Ethics and Dissemination: Ethical approval has been obtained from Keele University Ethical Review Panel (ref: MH-190017) and Khyber Medical University Ethical Review Board (ref: DIR-KMU-EB/ST/000648). The results of the STOPS+ trial will be reported in peer reviewed journals and academic conferences and disseminated to local stakeholders and policy makers.

Trial Registration information: https://doi.org/10.1186/ISRCTN93243890

For peer review only

STRENGTHS AND LIMITATIONS OF THIS STUDY

- This study aims to evaluate an approach that can be used for scaling up treatment for schizophrenia in resource poor settings and specifically addresses the gap in schizophrenia treatment.
- To the best of our knowledge this is first study that uses a DOTS (Directly Observed Therapy) approach translated from an infectious disorder i.e. Tuberculosis to a non-communicable disorder for scaling up the treatment for schizophrenia.
- The intervention involves primary care in treatment of schizophrenia in community, which is rarely done in LMIC.
- The trial is powered two primary outcomes (i) a clinical outcome measure – Global Assessment of Functioning and (ii) a process outcome measure – adherence to medication.
- The measurement of treatment adherence is based on a subjective measure i.e. reporting from patients and carers that cannot be objectively validated.

For peer review only

For peer review only - http://bmjopen.bmj.com/site/about/guidelines.xhtml

INTRODUCTION

Schizophrenia is one of the leading causes of years lived with disability, especially in low- and middle-income countries (LMICs) [1]. Existing evidence-based interventions for schizophrenia have been poorly implemented and most LMIC countries have large treatment gap for schizophrenia.

[2] The treatment gap represents the disparity between the true prevalence of a disorder and the treated proportion of individuals diagnosed [3]. Pakistan has one of the largest treatment gaps for schizophrenia, at an estimated 96%, this is contrast to an estimated median of 32.2% globally and 69% in LMICs.[4] [5].This treatment gap is attributed to a number of factors including poor treatment adherence with average non treatment adherence rates of about 50% in schizophrenia,[4] level of disability, a lack of primary care involvement and little to no access to treatment [5]. This is a particularly important factor in rural areas, where healthcare resources are often diminished in comparison to urban centres[6]. Whilst the cost of antipsychotic medication in Pakistan is relatively low, economic and logistical factors remain significant barriers to medication access [7] and the purchase of antipsychotic medication is often at the expense of other forms of essential out-of-pocket healthcare or even food.[8]

Reducing the treatment gap for schizophrenia in LMIC is critical. Medication non-adherence in patients with schizophrenia is associated with 2.8 times increased risk in hospital admission.[9] The risk of death in patients who receive even minimal treatment is significantly reduced compared with patients receiving no treatment at all [10]. Furthermore, untreated psychosis can at least double the costs of managing chronic co-morbid physical health conditions, leading to an increased stress on an already overburdened healthcare system.[11]

We previously reported a randomised controlled trial (RCT) of Supervised Treatment in Out-Patients for Schizophrenia (STOPS) in Peshawar, Pakistan [12]. STOPS was developed using the Medical Research Council framework for complex interventions [13] and was modelled on the Directly Observed Treatment Short-Course (DOTS) intervention to improve treatment adherence for people with Tuberculosis (TB). The DOTS approach involves a TB worker or a family member directly supervising the patient taking all medication and recording this [14]. DOTS is one of the most successful public health interventions and has been shown to be as highly effective in the community as it is in specialist clinics, which in addition to its accessibility and low cost, make it a highly appropriate intervention model for LMICs. Using the similar approach, STOPS intervention involved a family member of a person with schizophrenia observing and recording that medication had been taken. The family member is trained for this purpose. A previous evaluation of STOPS in a tertiary care setting showed that the STOPS significantly improved treatment adherence and functioning as well reducing patient symptoms.[11].

The original STOPS intervention was evaluated in a tertiary care setting by mental health professionals, with no primary care involvement, limiting its scaling up and generalisability. Through a programme of qualitative work with key stakeholders in Pakistan, including patients, carers, healthcare professionals and researchers, we adapted the STOPS model to

For peer review only - http://bmjopen.bmj.com/site/about/guidelines.xhtml

STOPS+ with the aim of scaling up the intervention and involving primary care in the management of schizophrenia.

Here, we outline the protocol for evaluating the STOPS+ in a real-world setting using a cluster RCT design.

METHODS AND ANALYSIS

Objectives

Primary objective

The primary objective is to evaluate the clinical effectiveness of the STOPS+ intervention in improving medication adherence and functioning in patients with schizophrenia compared to enhanced treatment as usual (ETAU) in primary care in Pakistan.

Secondary objectives

i) To evaluate the cost effectiveness of the STOPS+ intervention compared to ETAU;
ii) To assess the effectiveness of STOPS+ in reducing family/caregiver burden and stigma in the community;
iii) To evaluate if STOPS+ leads to physical health improvement in patients with schizophrenia;
iv) To investigate the implementation of STOPS+ in a primary care setting and the acceptability of STOPS+ for service users and healthcare providers, assessing the impact of STOPS+ in the wider healthcare system.

Study setting

The intervention will be implemented and evaluated in the Peshawar district of Khyber Pakhtunkhwa (KP). KP is one of four provinces in Pakistan located in the North West region with an estimated population of 20.7 million. Health expenditure in Pakistan is about 1% of the Gross Domestic Product (GDP) with no separate provision for mental health services. The primary health care (PHC) facilities in KP are provided by a network of Basic Health Units (BHUs), Civil Dispensaries (CDs), and Rural Health Centres (RHCs). Typically, each PHC facility consists of a primary care physician, a Multipurpose PHC technician (MT) and a Lady Health Worker (LHW).[15] Training PHC providers to manage mental health conditions and the incorporation of mental health services into primary care are both envisaged in current health policy, but have not yet been fully realised.[5]

Trial design

A cluster randomised controlled trial (RCT) will be used to investigate the efficacy of the STOPS+ intervention compared with Enhanced Treatment as Usual (ETAU) in people living with Schizophrenia. The cluster RCT design will help to evaluate the implementation of STOPS+ at the health care facility level and minimise the contamination of intervention and control arms.

For peer review only - http://bmjopen.bmj.com/site/about/guidelines.xhtml

INTERVENTION AND CONTROL

The control: Enhanced Treatment as Usual (ETAU)
Typically, in Pakistan, treatment for schizophrenia consists of accessing hospital based psychiatric services with little to no involvement of PHC and a limited provision of medication from the hospital pharmacy. ETAU provided in the control clusters will include the treatment received by patients' routine health care setting, which includes treatment provided by a psychiatrist in the outpatient clinics of the psychiatry department of the local hospital and brief counselling about the treatment and outcome of the disorder. Patients may be admitted to an inpatient unit at the hospital. This will be enhanced in the control cluster PHCs with training provided to PHC physicians in WHO Mental Health Gap Action Programme (mhGAP). (https://www.who.int/mental_health/mhgap/en/t) in addition to the regular and reliable provision of psychotropic drugs at the PHC. The patients will be informed that the antipsychotic medication is available in the PHC centre and they can access the centre for supply of medication. The PHC physician will have the option to liaise with the treating psychiatrist, as provided in the MhGAP guidelines.

STOPS+ intervention

The STOPS+ intervention will consist of the ETAU plus the following components;

1. Supervision by a trained family member for dispensing and administering medication
A family member who is living with the patient for a minimum of 6 months prior to recruitment to the trial will be nominated by the patient. The family member will dispense medication, observe that is has been taken correctly and record this information on a simple sheet of paper designed and used in the previous STOPS trial.[9] Family members will be reminded to administer the medication by an automated text message system, which will also request a response once the medication has been taken correctly. The text message reminders will be sent every day for the first three months after starting the treatment. If the family member does not have access to a mobile phone, a handset and training in using it will be provided. A Multi-Purpose technician (MPT) from each PHC centre will provide training to family members for the storage and administration of the medication as well as recording when the medication has been taken correctly.

2. Treatment for schizophrenia at PHC level supervised by mental health professionals
Ongoing management and monitoring of the patient's schizophrenia will take place at their PHC centre by the PHC physician, under the supervision of the treating psychiatrist with the help of the mobile technology platform SMART (Smartphone for Monitoring, Assessment and Remote Treatment). SMART will provide two-way communication between mental health care professionals. One month's supply of medication will be made available, via their PHC, to the family member. The automated messaging system will also remind the family member about collecting the following month's supply.

3. Monitoring the availability of essential psychotropic medication and their side effects

For peer review only - http://bmjopen.bmj.com/site/about/guidelines.xhtml

A kit to monitor the supply of medication will be used to monitor the availability and supply of psychotropic medication. This will consist of a) a cupboard dedicated specifically for the storage of psychotropic medication, b) a tracking sheet used to monitor the stock levels c) a simple checklist for monitoring side-effects of antipsychotics.

Assessment of barriers and facilitators of implementation of the intervention

The use of theory to inform the development of behaviour change interventions is strongly advocated by experts in the field. We have designed the process of implementation using theory developed for implementation and tested previously in UK Primary Care Practice [16,17]. We will evaluate the barriers and facilitators to implementation of the intervention using qualitative methods. We will hold at least four focus groups (with patients and carers, multipurpose technicians, traditional and faith healers and primary care clinicians) in which we will explore the barrier and facilitators to supervising the treatment of schizophrenia in the community, modification of the STOPS programme to STOPS+ for use in the community and the present status of schizophrenia care in the community

Patient and Public Involvement

STOPS+ is underpinned by culturally appropriate and context-bespoke patient and public community engagement and involvement at every stage of the project (intervention development, RCT and process evaluation). The STOPS+ intervention will be refined with input from the key stakeholder groups including patients, carers, health professionals, local councillors, community leaders such as religious leaders and community experts and policy makers. Small group meetings using local Jirgas (Jirga is traditional, local participatory and decision-making body which is well-established in Pathan culture and brings together local tribal, ethnic and religious leaders) will help in community engagement.

OUTCOMES AND MEASURES

Primary outcome measure

In view of the pragmatic nature of the trial, we will use two distinct primary outcomes:
(i) The primary clinical outcome will be the level of functioning, measured using the Global Assessment of Functioning (GAF) scale.[18] GAF is a commonly used tool in schizophrenia, particularly for the pragmatic trials.[19]
(ii) The primary process outcome will be adherence to treatment regimen. The adherence to treatment will be measured using a questionnaire adapted from Herz et al (2000).[20] This measure for assessing treatment adherence was successfully used in our previous STOPS study.[9]

Secondary outcomes:

The details of secondary outcomes and the standardised instruments for measuring these are given in Table 1. These instruments will measure secondary outcomes such as physical health outcomes, family/caregivers burden and internalised stigma.

For peer review only - http://bmjopen.bmj.com/site/about/guidelines.xhtml

Table 1. Description of outcome measures and instruments

	Domains measured	Measure
	Sociodemographics	Age<br>Gender<br>Years in education<br>Marital status<br>Work status<br>Mini-Neuropsychiatric Interview (MINI) for diagnosis of schizophrenia [21]
	Physical health	Body Mass Index (BMI)<br>Blood Pressure<br>Waist Circumference
**Primary outcomes**		
	Level of functioning	Global Assessment of Functioning (GAF) [18]
	Level of adherence	Treatment adherence rating scale [20]
**Secondary outcomes**		
	Mental state and psychiatric symptoms	Brief Psychiatric Rating Scale [22]
	Caregivers' burden	Family Burden Scale [23]
	Perceived stigma	Internalised Stigma of Mental Illness [24]
	Side effects of antipsychotic medication	Glasgow Antipsychotic Side-Effects Scale [25]
	Cost of care	Client Service Receipt Inventory [26]
	Quality of life and cost effectiveness	EuroQol (EQ5D) [27]
	Illness severity, improvement and response to treatment	Clinical Global Impression Scale [28]
	Drug use	DAST Drug Screening Tool [29]
	Depression	Patient Health Questionnaire [30]
	Suicide Ideation	Suicide Behaviours Questionnaire Revised [31]

Eligibility criteria

Patients will be eligible for inclusion if they;

For peer review only - http://bmjopen.bmj.com/site/about/guidelines.xhtml

a) have a diagnosis of schizophrenia (F20.9) or schizoaffective disorder (F25.9) based on the International Classification of Disease 10 (ICD-10) criteria assessed using the Mini-International Neuropsychiatric Interview [21]
b) are aged between 17-65 years
c) do not meet the criteria for remission as defined by the Remission in Schizophrenia Working Group [32]
d) have capacity and are able to give informed consent
e) Have a family member who is also willing to participate in the trial and supervise the treatment after training by the research team. .

Exclusion criteria will be kept to a minimum to ensure that the study can examine the real world implementation of the STOPS+. The patients who can't participate in the study due to one of following reason will be excluded I) a serious or unstable medical illness, II) evidence of learning disability, III) severe drug dependence requiring treatment and/or detoxification IV) pregnant or breast-feeding.

Duration of treatment and follow-up:

Both groups will be followed up for 12 months after recruitment in the study. The follow up using face-to-face assessments will be carried out by trained research assistants in a health facility which is not part of participating PHCs to preserve the blindness of clusters. All of the outcomes will be measured at baseline, at 6 months and at 12 months by research assistants.

Participant Identification

Participants will be identified from two possible sources i) patients living in the area of the participating PHCs and presenting at psychiatry outpatient services of the hospitals in the area (ii) potentially eligible patients identified by the primary health care workers during community engagement or attending the PHCs. Eligible participants will be informed about the trial orally by the PHC staff and written information will be provided in the local language (Urdu). Those interested in taking part will be asked to give written informed consent. For participants who cannot read and write, witnessed oral consent and a thumb print in lieu of a signature will be used. The witness will not be a member of the research team. The procedure has been used in our previous trials in the area.[33]

Randomisation and allocation concealment

Randomisation will occur at the PHC level, where PHCs will be allocated at a ratio of 1:1 to either deliver the STOPS+ intervention or ETAU. Twenty-four PHCs will be recruited to the study, 12 will be randomised to deliver ETAU and 12 will be randomised to deliver the STOPS+ programme. PHC randomisation will be stratified by urban/rural setting. Randomisation will be carried out by an independent statistician at the Khyber Medical University (KMU), Peshawar by using a remote computer generated random sequence. Randomisation and will be based on the list of primary care centres in Peshawar, provided by the health department of KP. The urban/rural classification is determined as per the Peshawar district council. It is not possible to blind study participants from their treatment arm allocation. However, the assessment team that conducts the baseline and follow up

For peer review only - http://bmjopen.bmj.com/site/about/guidelines.xhtml

assessments (6 and 12 months) will be masked to the treatment arm of the cluster. The outcome measures will be administered by trained research assessment teams (outcome assessors), who are independent of the intervention team and the team involved in consenting and screening. The outcome assessment will not be carried out at any participating PHC. The assessment teams will be told that they are evaluating two interventions and that there is genuine equipoise about which one is better. Study participants will be instructed to not discuss the PHC they are attending or the treatment details. During all assessments, the primary outcome measures (treatment adherence scale and GAF) will be completed first to minimise the risk of bias in the event of unmasking and, if it occurs, the point of unmasking will be recorded. Sensitivity analyses will be carried out to assess the effect of unmasking on the primary outcomes. The data linking each PHC with treatment allocation status is kept separate from the outcome dataset until the time of the final analysis. The trial statistician will be blind to allocation status.

Screening, Data collection and follow up

After identification of potentially eligible patients, they will first be orally informed about the trial by one of the health workers in the local Pushto language (MT in the community or a health professional in one of the health facilities mentioned above). They will be asked whether they agree that a member of the research team will provide them with further information about the research. If permission is given, a research assistant will meet with the patient and will request informed consent. The informed consent for participation in the study will be taken within one week of the first contact. Participants will be free to decline to participate or withdraw at any time without their routine health care being affected. Before taking part in any interviews, oral and written information about the study and its purpose will be provided to respondents in the local language.

After obtaining informed consent the research assistant will decide whether the patient meets the inclusion criteria and have a family member who is willing to supervise the treatment as specified in the STOPS+ procedures. If the patient is not eligible from the screening assessment the reason for ineligibility will be explained to the patient and they will be signposted for further advice and to continue to receive treatment at their routine health facility. Eligible patients will be provided with a further participant information sheet about the trial. Additionally, for those in the intervention arm, the nominated relative will receive a participant information sheet about the trial and about the tasks required in supervising the treatment If nominated family member is available with the patient, this information will be provided at the same time. If the family member is not available, patients and the family member will be asked to visit the health facility again within one week. The patient and the relative will each be given the opportunity to ask any questions about the trial. The information sheets are provided in Urdu. For illiterate patients the research assistants will explain the same information in local Pushto language or in Urdu. Following the informed consent, the baseline data will be collected.

Sample size calculation

A mean difference of 6 on the primary clinical outcome measure (GAF) with a pooled study standard deviation of 15 was observed in our previous trial in the same setting.[12] A recent indicated minimum clinically important difference for the GAF in schizophrenia patients has

For peer review only - http://bmjopen.bmj.com/site/about/guidelines.xhtml

been given as a mean difference of 4).[34] A total sample size of 526 i.e. 263 patients in each arm (recruited across 24 PHCs, with an average cluster size of 22 patients) has over 90% power to detect a mean difference of 6 and 85-90% power to detect a more modest mean difference of 4 in the GAF given a two-tailed significance level of 0.05 and based on the following parameter assumptions: i) baseline-outcome correlation of 0.5, repeated measures correlation of 0.7 [35] and loss to follow up of 20% (patient-level parameters); ii) intracluster correlation coefficient of 0.01 [36] and coefficient of variation in cluster sizes of 0.65 (PHC-level parameters).[37] The primary endpoint for the GAF is the 'average' GAF across 6 and 12 months follow up.

This sample size has 90% power to detect at least a 20% absolute difference in the treatment adherence rate between the intervention arm compared to the ETAU arm at 6 and/or 12 months follow up based on two-tailed alpha of 0.025 (to account for multiple testing in respect of 6 and 12 month testing). This is based on an observed ≈20% higher level of adherence within the intervention compared to ETAU arms in the original STOPS trial at 3 and 12 months follow up,[11] and further assuming the design effect PHC-level parameters as noted in (ii) above. Thus, the study is adequately powered to detect significant differences (at the level of 2-tailed alpha of 0.05 in each case) across the two distinct primary outcomes: i) the primary clinical outcome (GAF) and ii) the primary process outcome (medication adherence).

Trial management and monitoring

The STOPS+ trial will be monitored in line with the protocol and the trial standard operating procedures. An independent Trial Steering Committee (TSC) which will have service user representatives will monitor trial progress. The TSC is empowered to independently review the ethical and data management procedures. A Data and Safety Monitoring Board (DSMB) will be convened to ensure the safety of participants and the integrity of the data. The PHCs in the trial will receive training around safety reporting and additionally, participants and their family members will be informed of how to report concerns and safety issues, whilst they are participating in the trial. The Project Management Committee (PMC) will be responsible for setting up the trial, ongoing management and monitoring, promotion of the study, training and the interpretation of the results.

Safety and Adverse events reporting

All adverse events and serious adverse events (SAEs) that are reported by the participant, family member or identified by the trial team or PHC staff members during the trial will be recorded and reported following trial safety reporting procedures. Related and unexpected SAEs will be reported to the local Research Ethics Committee (REC), DSMB and TSC within 15 days. The DSMB will review all safety data and make any decisions regarding early termination of the study in line with their respective terms of reference.

Data management

All data will be managed in line with the protocol and will be stored securely following the FKMU and Keele University standard policy on data storage and confidentiality. Individual participant data will be pseudonymised through the use of a unique study ID and will be

For peer review only - http://bmjopen.bmj.com/site/about/guidelines.xhtml
For peer review only

stored securely and separately from any identifiable data. Access to data will be restricted to members of the research team. Outcome data will be entered into an electronic database which will be stored on an encrypted secure network at KMU requiring a password to access. Data entry will be quality checked and coded using standard processes.

Statistical analysis

Statistical analysis will be performed blind to cluster allocation. Primary analysis will be on an intention-to-treat (ITT) basis, with all participants analysed in their cluster. Between-group differences for both primary outcome measures (GAF and medication adherence) will be analysed using longitudinal mixed models that accommodate clustering at the (upper/hierarchical) practice level, as well as repeated outcome measurements across 3 time points (baseline, 6 and 12 months) per participant. i.e. linear/logistic mixed models for numerical and categorical outcomes, respectively, including PHC and participant as random factors [37]. These models will be used to quantify the absolute between group difference in the primary clinical outcome (GAF) and odds ratio (and extracted absolute difference) for the primary process outcome (% adherence) – using linear and logistic models, respectively. For the GAF, the primary endpoint is the 'average' GAF over 6 and 12 months using available and modelled data to estimate the combined summary average mean difference in GAF between treatment arms over the 6 and 12 months timeline; statistical significance in this case being given against two-tailed p=0.05. Though the 'average' is the single primary endpoint for the GAF, we will also evaluate the treatment-by-time interaction to give estimates of between-arm mean differences at the individual 6 and 12 months follow-up time points (secondary endpoints).

For medication adherence, between-group comparison at both 6 and 12 months are primary (derived through treatment-by-time interaction within the generalised mixed model) – hence, the requirement to take into account the multiple-testing by assessing significance against a more conservative two-tailed p=0.025. Estimates of between-group mean differences will be given including 95% confidence intervals and p-values. The mixed models (detailed above) will be adjusted for age, gender, baseline GAF score and corresponding baseline score of the outcome being measured as individual-patient level covariates, PHC-level characteristics (PHC size), stratification variable (rural/urban) and a random effect for the PHC. Mixed model analysis fulfils the ITT principle with missing data being accounted for under the missing at random (MAR) assumption.

Cost effectiveness

The cost of STOPS+ will be evaluated against ETAU using the Service Receipt Inventory (SRI),[38] which has been used in previous trials in Pakistan.[21] This will include the cost of primary and outpatient care visits, inpatient admissions, drug regimens, diagnostic tests, travel time, transport costs and lost days from work. Costs will be subsequently linked to outcomes in order to establish whether STOPS+ represents a cost-effective use of resources (e.g. whether the additional cost of supervision by the relative are offset by the reduced hospital care and/or improvements in the clinical outcome – GAF and QALY). Cost-effectiveness planes and acceptability curves will evaluate the uncertainty in estimates as well as the probability of STOPS+ being cost-effective across a range of willingness-to-pay thresholds. Adjusted analysis will be carried to accommodate clustering of PHCs.

For peer review only - http://bmjopen.bmj.com/site/about/guidelines.xhtml

Ethics and dissemination

Ethical approval to conduct this trial have been obtained from Keele University Ethical review Panel (reference: MH-190017) and Khyber Medical University Ethical Review Board (ref: DIR-KMU-EB/ST/000648). Subsequently, recruitment began on the 1st of November 2019. Results will be disseminated through peer reviewed journals and academic conferences. The trial is being conducted in accordance with the International Conference on Harmonisation/Good Clinical Practice (ICHGCP) Guidelines (1996) which are consistent with principles of Declaration of Helsinki (1996) Ethical Principles for Medical Research Involving Human Subjects.

Process evaluation

Patients and relatives, primary care physicians and multipurpose technician from participating PHCs in both arms will be invited to take part in a semi-structured interview to assess the acceptability of intervention and its implementation in the health system.[39] These interviews will last approximately 30-40 minutes and will be audio recorded with permission. A minimum of 20 individual semi-structured interviews [40] will be conducted (continuing until data saturation is reached)[41,42] with patients and their relatives to explore a) the acceptability of the STOPS+ intervention; b) the experience/burden of the training and support for the patient's family member; c) barriers and facilitators to medication adherence and overall satisfaction with the intervention and trial participation. A minimum of 10 interviews will be conducted with PHC physicians. In addition, we will conduct interviews with at least 5 decision-makers with responsibilities for developing or implementing health policy in the directorate of health in order to capture their perceptions about the feasibility, the benefits, challenges and acceptability of STOPS+ in the health system. Participants for these interviews will be purposively sampled to stratify by gender and urban/rural setting. A semi-structured topic guide will be used to facilitate interviews. Informed consent will be obtained prior to the start of the interview and will include consent for the use of direct pseudonymised quotes.

An inductive, exploratory framework will be adopted using thematic analysis based on the principles of grounded theory.[43,44] A sample of early transcripts will be independently coded by the research team and a coding framework agreed upon. This framework will then be applied to subsequent coding. Coded data will be analysed independently by qualitative study leads at Keele and KMU to develop categories and themes to be discussed in wider research team meetings. Constant comparison will be used to explore connections within and across transcripts and codes, highlighting consistencies and variation.[45] Analysis will follow an iterative process, with emergent findings used to further refine topic guides for subsequent interviews.

DISCUSSION

People suffering from schizophrenia represent one of the most disadvantaged groups in low- and middle-income countries facing a wide mental health treatment gap. A number of psychosocial interventions have been evaluated in RCTs and there is consensus that the

For peer review only

For peer review only - http://bmjopen.bmj.com/site/about/guidelines.xhtml

treatment of schizophrenia should combine anti-psychotic medication and psychosocial interventions.[46] However there is limited evidence how these interventions can be implemented in real world settings.

The proposed study adopts an implementation approach addressing systemic barriers to reduce treatment gap for schizophrenia in the community. The STOPS+ intervention aims to address three major implementation barriers (i) improving supply of antipsychotic medication at primary care level (ii) involving primary care workers in the treatment of schizophrenia (iii) improving treatment adherence by involving family members in supervising treatment. The STOPS+ approach therefore goes beyond most psychosocial interventions evaluated in treatment of schizophrenia in LMIC countries [47, 48] in addressing the health system, community and service users related factors to improve access to treatment.
A unique aspect of the trial is, that it is powered for process and clinical outcomes. These two primary outcomes are: (i) a clinical outcome measure – Global Assessment of Functioning and (ii) a process outcome measure – adherence to medication. This combined with a process evaluation carried out after completion of the cluster RCT will provide much needed evidence on the implementation of community-based interventions for schizophrenia in LMIC countries settings. The WHO Mental Health Action Plan (2013-2020) requires that there is an absolute increase of 20% in service coverage for severe mental disorders.[48] The results of the trial may help to understand the processes and implementation strategies to achieve that goal and could provide an effective public health intervention for schizophrenia.[49]

A major strength of the STOPS intervention is that it is based on an infectious disease model that has been successfully used in improving treatment adherence for Tuberculosis i.e. DOTS (http://www.searo.who.int/tb/topics/what_dots/en/) as a public health intervention. The DOTS (Directly Observed Treatment Short course) model is currently being used in most LMIC countries, including the settings of this trial. This can potentially help in integration of the care for a non-communicable chronic disorder like schizophrenia in the public health systems of LMIC countries.

Competing interests statement: SF in the past 3 years has received honoraria and speaker's fees from Otsuka, Lundbeck and Sunovian pharma and also research funding from Sunovian Pharmaceutical. MMuH and MFK received funding from different pharmaceuticals to attend scientific meetings.

Acknowledgements: Krysia Dziedzic is an NIHR Senior Investigator. The views expressed in this paper are those of the authors and not necessarily those of the NHS, NIHR, or the Department of Health and Social Care.

For peer review only - http://bmjopen.bmj.com/site/about/guidelines.xhtml

References

1 GBD 2017 Disease and Injury Incidence and Prevalence Collaborators. Global, regional, and national incidence, prevalence, and years lived with disability for 354 diseases and injuries for 195 countries and territories, 1990–2017: a systematic analysis for the Global Burden of Disease Study 2017. *The Lancet. 2018;**392**(10159)*:1789-1858

2 Charlson FJ, Ferrari AJ, Santomauro DJ, et al. Global Epidemiology and Burden of Schizophrenia: Findings From the Global Burden of Disease Study 2016. *Schizophrenia Bulletin* 2018;**44**(6):1195-1203.

3 Patel V, Maj M, De Silva MJ, Koschorke M & Prince M. Estimating the treatment gap for mental disorders: a WPA survey. *World Psychiatry* 2010:9(3) 169-176

4 Lora A, Kohn R, Levav I, et al. Service availability and utilization and treatment gap for schizophrenic disorders: a survey in 50 low- and middle-income countries. *Bull World Health Organ* 2012;**90**(1):47-54B.

5 De Silva MJ, Lee L, Fuhr DC, Rathod S, Chisholm D, Schellenberg J & Patel V. Estimating coverage of mental health programs: a systematic review. *International Journal of Epidemiology* 2014;43(2) 341-353.

6 Kagstrom A, Alexova A, Tuskova E, Csajbók Z, Schomerus G, Formanek T, Mladá K, Winkler P, Cermakova P. The treatment gap for mental disorders and associated factors in the Czech Republic. *European Psychiatry* 2019;59,37-43.

7 Large M, Farooq S, Nielssen O, et al. Relationship between gross domestic product and duration of untreated psychosis in low- and middle-income countries. *Br J Psychiatry* 2008;**193**(4):272-278.

8 Farooq S, Burns J, Sumathipala A, et al. A Global Mental Health Fund for the treatment of Serious Mental Illness in Low Income and Middle Income Countries. *The Lancet Psychiatry 2016;***3**(6):182-183.

9 Weiden PJ and Olfson M. Cost of relapse in schizophrenia. *Schizophr Bull* 1995;**21**(3):419-429.

10 Farooq S, Large M, Nielssen O, et al. The relationship between the duration of untreated psychosis and outcome in low-and-middle income countries: a systematic review and meta analysis. *Schizophr Res* 2009;**109**(1-3):15-23.

11 McDaid D and Park A-L. Counting all the costs: the economic impact of comorbidity. *Karger Publishers* 2014.

12 Farooq S, Nazar Z, Irfan M, et al. Schizophrenia medication adherence in a resource-poor setting: randomised controlled trial of supervised treatment in out-patients for schizophrenia (STOPS). *Br J Psychiatry* 2011;**199**(6):467-472.

For peer review only - http://bmjopen.bmj.com/site/about/guidelines.xhtml

13 Campbell M, Fitzpatrick R, Haines A, et al. Framework for design and evaluation of complex interventions to improve health. *BMJ* 2000;**321**(7262):694-696.

14 World Health Organization. Communicable Diseases Cluster. (1999). What is DOTS? : a guide to understanding the WHO-recommended TB control strategy known as DOTS. World Health Organization.

15 Government of Khyber Pakhtunkhwa. Mental Health And Psychosocial Support: Strategy And 5-Year Multi-Sectoral Action Plan For Khyber Pakhtunkhwa (2017-2022). *Government of Khyber Pakhtunkhwa* 2017.

16 Grol R, Wensing M, Eccles M: Improving Patient Care - The Implementation of Change in Clinical Practice. 2004, London: Elsevier

17 Michie S, van Stralen MM, West R: The behaviour change wheel: a new method for characterising and designing behaviour change interventions. Implement Sci. 2011, 6: 42-10.1186/1748-5908-6-42.

18 American Psychiatric Association. DSM-IV-TR: Diagnostic and statistical manual of mental disorders, text revision. Washington, DC: American Psychiatric Association 2000;**75**.

19 Crawford MJ, Killaspy H, Barnes TRE, et al. Group art therapy as an adjunctive treatment for people with schizophrenia: multicentre pragmatic randomised trial. *BMJ* 2012;344:e846

20 Herz MI, Lamberti S, Mintz J, et al. A program for relapse prevention in schizophrenia. A controlled study. *Arch Gen Psychiatry* 2000;57:277–283.

21 Sheehan DV, Lecrubier Y, Sheehan KH, et al. The Mini-International Neuropsychiatric Interview (M.I.N.I.): the development and validation of a structured diagnostic psychiatric interview for DSM-IV and ICD-10. *J Clin Psychiatry* 1998;**59**(Suppl 20):22-33.

22 Overall, JE, & Gorham, DR. The Brief Psychiatric Rating Scale. *Psychological Reports* 1962;10(3):799–812.

23 Pai S & Kapur RL. The burden on the family of a psychiatric patient: development of an interview schedule. *Br J Psychiatry* 1981;**138**:332-335.

24 Lysaker PH, Davis LW, Warma DM, Strasburger A & Beattie N. Stigma, social function and symptoms in schizophrenia and schizoaffective disorder: associations across 6 months**.** *Psychiatry Research* 2007;14:89-95.

25 Waddell L, Taylor M. A new self-rating scale for detecting atypical or second-generation antipsychotic side effects. *Journal Psychopharmacology* 2008; 22:238

26 Chisholm D, Knapp MR, Knudsen HC, Amaddeo F, Gaite L, van Wijngaarden B. Client Socio-Demographic and Service Receipt Inventory--European Version: development of an instrument for international research. EPSILON Study 5. European Psychiatric Services:

For peer review only

For peer review only - http://bmjopen.bmj.com/site/about/guidelines.xhtml

Inputs Linked to Outcome Domains and Needs.
Br J Psychiatry Suppl. 2000;(39):28-33.

27 The EuroQol Group (1990). EuroQol-a new facility for the measurement of health-related quality of life. *Health Policy* 1990;16(3):199-208.

28 Guy W (ed). ECDEU Assessment Manual for Psychopharmacology. Rockville, MD: US Department of Heath,Education, and Welfare Public Health Service Alcohol, Drug Abuse, and Mental Health Administration, 1976 Busner J, Targum SD. The clinical global impressions scale: applying a research tool in clinical practice. *Psychiatry (Edgmont)* 2007;4(7):28-37

29 Skinner HA. The Drug Abuse Screening Test. *Addictive Behavior* 1982;*7*(4): 363–371.

30 Kroenke K, Spintzer RL, Williams JB. The PHQ-9: validity of a brief depression severity measure. Journal of General Internal Medicine 2001;16(9):606-613

31 Osman A, Bagge CL, Gutierrez PM, Konick LC, Kopper BA, Barrios FX., The Suicide Behaviours Questionnaire-Revised (SBQ-R): Validation with clinical and nonclinical samples. *Assessment*. 2001 (5):443-54

32 Andreasen NC, Carpenter WT Jr, Kane JM, et al. Remission in schizophrenia: proposed criteria and rationale for consensus. *Am J Psychiatry* 2005;**162**(3):441-449.

33 Rahman A, Hamdani SU, Awan NR, et al. (2016). Effect of a Multicomponent Behavioural Intervention in Adults Impaired by Psychological Distress in a Conflict-Affected Area of Pakistan: A Randomized Clinical Trial. *JAMA* 2016;316(24):2609-2617.

34 Amri I, Millier A, Toumi M. PMH2 - Minimum Clinically Important Difference in the Global Assessment Functioning in Patients with Schizophrenia. *Value in Health* 2014;17:A765–A766.

35 Vickers AJ. How many repeated measures in repeated measures designs? Statistical issues for comparative trials. *BMC Med Res Methodol* 2003;**3**:22.

36 Adams G, Gulliford MC, Ukoumunne OC, et al. Patterns of intra-cluster correlation from primary care research to inform study design and analysis. *J Clin Epidemiol* 2004;**57**(8):785-94.

37 Eldridge SM, Ashby D and Kerry S. Sample size for cluster randomized trials: effect of coefficient of variation of cluster size and analysis method. *Int J Epidemiol* 2006;35(5):1292-300.

38 Chisholm D, Knapp MR, Knudsen HC, et al. Client socio-demographic and service receipt inventory--european version: development of an instrument for international research. EPSILON study 5. European psychiatric services: inputs linked to outcome domains and needs. *Br J Psychiatry Suppl* 2000;39:s28–33.

39 O'Cathain A. A practical guide to using qualitative research with randomized controlled trials. Oxford University Press 2018.

For peer review only - http://bmjopen.bmj.com/site/about/guidelines.xhtml

40 Malterud K, Siersma VD and Guassora AD. Sample size in qualitative interview studies: guided by information power. *Qualitative health research* 2016;26(13):1753-1760.

41 Saunders B, Sim J, Kingstone T, et al 2018. Saturation in qualitative research: exploring its conceptualization and operationalization. *Quality & quantity* 2018;52(4):1893-1907.

42 Sim J, Saunders B, Waterfield J, et al. Can sample size in qualitative research be determined a priori?. *International Journal of Social Research Methodology 2018;*21(5):619-634.

43 Glaser BG and Strauss AL. Discovery of grounded theory: Strategies for qualitative research. Routledge 2017.

44 Braun V, Clarke V, Hayfield N, et al. Thematic analysis. Handbook of research methods in health social sciences. Singapore: Springer 2018:1-18.

45 Nowell LS, Norris JM, White DE, et al. Thematic analysis: Striving to meet the trustworthiness criteria. *International Journal of Qualitative Methods* 2017;16(1): p.1609406917733847.

46 Asher L, Patel V, De Sliva MJ. Community-based psychosocial interventions for people with schizophrenia in low and middle-income countries: systematic review and meta-analysis. *BMC Psychiatry* 2017;**17**:355.

47 Xu DR, Xiao S, He H, et al. Lay health supporters aided by mobile text messaging to improve adherence, symptoms, and functioning among people with schizophrenia in a resource-poor community in rural China (LEAN): A randomized controlled trial. *PLoS Med* 2019;**16**(4):e1002785.

48 Kaddick H, Horney B, McKenzie J, Tilley H. Investing in mental health in low and middle income countries. Overseas Development Institute, London 2016.

49 Farooq, S. Public health and physician focused strategies to improve medication adherence in psychotic disorders. *World Psychiatry* 2013;12(3):238-239.

Authors' contributions: TAS wrote first draft. All authors contributed to the conceptualisation and the design of the study. SF obtained the funding for the study, conceived and developed the intervention and study design, and led creation of the team. MFK, ZuH, MMuH, AA and US secured the study sites and contributed to design and development of intervention, community engagement and qualitative studies leading to modification of intervention. LD contributed to design and led qualitative studies for intervention development and process evaluation. US, TAS, ZuH and MER contributed to writing ethics applications and obtaining the ethics approvals. ML contributed significantly to the study design, analysis plan and economic evaluation part of the protocol. AR,LD, KD, CDM provided critical review and contributed to the design of the study. All authors read and revised the initial manuscript and approved the final version.

For peer review only - http://bmjopen.bmj.com/site/about/guidelines.xhtml

Funding statement – This work is supported by the Medical Research Council UK, as part of their Global Alliance for Chronic Disease programme grant number MR/S00243X/1.

For peer review only

For peer review only - http://bmjopen.bmj.com/site/about/guidelines.xhtml

SPIRIT

STANDARD PROTOCOL ITEMS: RECOMMENDATIONS FOR INTERVENTIONAL TRIALS

SPIRIT 2013 Checklist: Recommended items to address in a clinical trial protocol and related documents*

Section/item	Item No	Description	Addressed on page number
**Administrative information**			
Title	1	Descriptive title identifying the study design, population, interventions, and, if applicable, trial acronym	______8_______
Trial registration	2a	Trial identifier and registry name. If not yet registered, name of intended registry	______39_______
	2b	All items from the World Health Organization Trial Registration Data Set	_______*______
Protocol version	3	Date and version identifier	_______1______
Funding	4	Sources and types of financial, material, and other support	_______5______
Roles and responsibilities	5a	Names, affiliations, and roles of protocol contributors	_______5,6,7____
	5b	Name and contact information for the trial sponsor	_______5*______
	5c	Role of study sponsor and funders, if any, in study design; collection, management, analysis, and interpretation of data; writing of the report; and the decision to submit the report for publication, including whether they will have ultimate authority over any of these activities	______na_______
	5d	Composition, roles, and responsibilities of the coordinating centre, steering committee, endpoint adjudication committee, data management team, and other individuals or groups overseeing the trial, if applicable (see Item 21a for data monitoring committee)	______na_______

For peer review only

**Introduction**			
Background and rationale	6a	Description of research question and justification for undertaking the trial, including summary of relevant studies (published and unpublished) examining benefits and harms for each intervention	_______10,11____
	6b	Explanation for choice of comparators	________11______
Objectives	7	Specific objectives or hypotheses	_______12_______
Trial design	8	Description of trial design including type of trial (eg, parallel group, crossover, factorial, single group), allocation ratio, and framework (eg, superiority, equivalence, noninferiority, exploratory)	________?______
**Methods: Participants, interventions, and outcomes**			
Study setting	9	Description of study settings (eg, community clinic, academic hospital) and list of countries where data will be collected. Reference to where list of study sites can be obtained	_______17,18____
Eligibility criteria	10	Inclusion and exclusion criteria for participants. If applicable, eligibility criteria for study centres and individuals who will perform the interventions (eg, surgeons, psychotherapists)	_______18_______
Interventions	11a	Interventions for each group with sufficient detail to allow replication, including how and when they will be administered	_____14,15,16,17_
	11b	Criteria for discontinuing or modifying allocated interventions for a given trial participant (eg, drug dose change in response to harms, participant request, or improving/worsening disease)	_____26_______
	11c	Strategies to improve adherence to intervention protocols, and any procedures for monitoring adherence (eg, drug tablet return, laboratory tests)	_______16, 34____
	11d	Relevant concomitant care and interventions that are permitted or prohibited during the trial	_______26_______
Outcomes	12	Primary, secondary, and other outcomes, including the specific measurement variable (eg, systolic blood pressure), analysis metric (eg, change from baseline, final value, time to event), method of aggregation (eg, median, proportion), and time point for each outcome. Explanation of the clinical relevance of chosen efficacy and harm outcomes is strongly recommended	12,13,21,22,23,24
Participant timeline	13	Time schedule of enrolment, interventions (including any run-ins and washouts), assessments, and visits for participants. A schematic diagram is highly recommended (see Figure)	_______9_______

For peer review only - http://bmjopen.bmj.com/site/about/guidelines.xhtml

Sample size	14	Estimated number of participants needed to achieve study objectives and how it was determined, including clinical and statistical assumptions supporting any sample size calculations	___26,27_______
Recruitment	15	Strategies for achieving adequate participant enrolment to reach target sample size	______27_______

Methods: Assignment of interventions (for controlled trials)

Allocation:

Sequence generation	16a	Method of generating the allocation sequence (eg, computer-generated random numbers), and list of any factors for stratification. To reduce predictability of a random sequence, details of any planned restriction (eg, blocking) should be provided in a separate document that is unavailable to those who enrol participants or assign interventions	______19_______
Allocation concealment mechanism	16b	Mechanism of implementing the allocation sequence (eg, central telephone; sequentially numbered, opaque, sealed envelopes), describing any steps to conceal the sequence until interventions are assigned	_______8______
Implementation	16c	Who will generate the allocation sequence, who will enrol participants, and who will assign participants to interventions	_______19______
Blinding (masking)	17a	Who will be blinded after assignment to interventions (eg, trial participants, care providers, outcome assessors, data analysts), and how	____19,20______
	17b	If blinded, circumstances under which unblinding is permissible, and procedure for revealing a participant's allocated intervention during the trial	_____________

Methods: Data collection, management, and analysis

Data collection methods	18a	Plans for assessment and collection of outcome, baseline, and other trial data, including any related processes to promote data quality (eg, duplicate measurements, training of assessors) and a description of study instruments (eg, questionnaires, laboratory tests) along with their reliability and validity, if known. Reference to where data collection forms can be found, if not in the protocol	__21,22,23,24___
	18b	Plans to promote participant retention and complete follow-up, including list of any outcome data to be collected for participants who discontinue or deviate from intervention protocols	______na_______

Data management	19	Plans for data entry, coding, security, and storage, including any related processes to promote data quality (eg, double data entry; range checks for data values). Reference to where details of data management procedures can be found, if not in the protocol	___32,33,34_____
Statistical methods	20a	Statistical methods for analysing primary and secondary outcomes. Reference to where other details of the statistical analysis plan can be found, if not in the protocol	______28,29____
	20b	Methods for any additional analyses (eg, subgroup and adjusted analyses)	_______29,30___
	20c	Definition of analysis population relating to protocol non-adherence (eg, as randomised analysis), and any statistical methods to handle missing data (eg, multiple imputation)	______29_______
**Methods: Monitoring**			
Data monitoring	21a	Composition of data monitoring committee (DMC); summary of its role and reporting structure; statement of whether it is independent from the sponsor and competing interests; and reference to where further details about its charter can be found, if not in the protocol. Alternatively, an explanation of why a DMC is not needed	______34_______
	21b	Description of any interim analyses and stopping guidelines, including who will have access to these interim results and make the final decision to terminate the trial	______30_______
Harms	22	Plans for collecting, assessing, reporting, and managing solicited and spontaneously reported adverse events and other unintended effects of trial interventions or trial conduct	___35,36,45_____
Auditing	23	Frequency and procedures for auditing trial conduct, if any, and whether the process will be independent from investigators and the sponsor	___33,34,35,36__
**Ethics and dissemination**			
Research ethics approval	24	Plans for seeking research ethics committee/institutional review board (REC/IRB) approval	______39_______
Protocol amendments	25	Plans for communicating important protocol modifications (eg, changes to eligibility criteria, outcomes, analyses) to relevant parties (eg, investigators, REC/IRBs, trial participants, trial registries, journals, regulators)	______na_______

For peer review only - http://bmjopen.bmj.com/site/about/guidelines.xhtml

Consent or assent	26a	Who will obtain informed consent or assent from potential trial participants or authorised surrogates, and how (see Item 32)	_______20________
	26b	Additional consent provisions for collection and use of participant data and biological specimens in ancillary studies, if applicable	_______na________
Confidentiality	27	How personal information about potential and enrolled participants will be collected, shared, and maintained in order to protect confidentiality before, during, and after the trial	_____31,32,33_____
Declaration of interests	28	Financial and other competing interests for principal investigators for the overall trial and each study site	_______39________
Access to data	29	Statement of who will have access to the final trial dataset, and disclosure of contractual agreements that limit such access for investigators	_______40________
Ancillary and post-trial care	30	Provisions, if any, for ancillary and post-trial care, and for compensation to those who suffer harm from trial participation	________39_______
Dissemination policy	31a	Plans for investigators and sponsor to communicate trial results to participants, healthcare professionals, the public, and other relevant groups (eg, via publication, reporting in results databases, or other data sharing arrangements), including any publication restrictions	_____40,41_______
	31b	Authorship eligibility guidelines and any intended use of professional writers	________*_______
	31c	Plans, if any, for granting public access to the full protocol, participant-level dataset, and statistical code	________*_______
**Appendices**			
Informed consent materials	32	Model consent form and other related documentation given to participants and authorised surrogates	_______*________
Biological specimens	33	Plans for collection, laboratory evaluation, and storage of biological specimens for genetic or molecular analysis in the current trial and for future use in ancillary studies, if applicable	_______na________

*It is strongly recommended that this checklist be read in conjunction with the SPIRIT 2013 Explanation & Elaboration for important clarification on the items. Amendments to the protocol should be tracked and dated. The SPIRIT checklist is copyrighted by the SPIRIT Group under the Creative Commons "Attribution-NonCommercial-NoDerivs 3.0 Unported" license.

For peer review only - http://bmjopen.bmj.com/site/about/guidelines.xhtml

BMJ Open

Supervised Treatment in Outpatients for Schizophrenia Plus (STOPS+): Protocol for a cluster randomised trial of a community-based intervention to improve treatment adherence and reduce the treatment gap for schizophrenia in Pakistan

Journal:	*BMJ Open*
Manuscript ID	bmjopen-2019-034709.R2
Article Type:	Protocol
Date Submitted by the Author:	22-May-2020

Complete List of Authors:	Shepherd, Thomas; Keele University, School of Primary, Community and Social Care<br>Ul-Haq, Zia; Khyber Medical University, Institute of Public Health & Social Sciences<br>Ul-Haq, Mian; Lady Reading Hospital<br>Khan, Muhammad Firaz; Lady Reading Hospital<br>Afridi, Adil; Lady Reading Hospital<br>Dikomitis, Lisa; Keele University, School of Primary, Community and Social Care; Keele University, School of Medicine<br>Robinson, Michelle; Keele University, School of Primary, Community and Social Care<br>Lewis, Martyn; Keele University, School of Primary, Community and Social Care<br>Rahman, Atif; University of Liverpool, Child Mental Health Unit<br>Dziedzic, Krysia; Keele University, School of Primary, Community and Social Care<br>Saeed, Umaima; Khyber Medical University, Institute of Public Health & Social Sciences<br>Awan, Naila; Lady Reading Hospital<br>Mallen, Christian; Keele University, School of Primary, Community and Social Care; Midlands Partnership Foundation Trust<br>Farooq, Saeed; Keele University, School of Primary, Community and Social Care; Midlands Partnership Foundation Trust

**Primary Subject Heading**:	Global health
Secondary Subject Heading:	Mental health
Keywords:	MENTAL HEALTH, Clinical trials < THERAPEUTICS, PUBLIC HEALTH, PSYCHIATRY, Schizophrenia & psychotic disorders < PSYCHIATRY

SCHOLARONE™
Manuscripts

I, the Submitting Author has the right to grant and does grant on behalf of all authors of the Work (as defined in the below author licence), an exclusive licence and/or a non-exclusive licence for contributions from authors who are: i) UK Crown employees; ii) where BMJ has agreed a CC-BY licence shall apply, and/or iii) in accordance with the terms applicable for US Federal Government officers or employees acting as part of their official duties; on a worldwide, perpetual, irrevocable, royalty-free basis to BMJ Publishing Group Ltd ("BMJ") its licensees and where the relevant Journal is co-owned by BMJ to the co-owners of the Journal, to publish the Work in this journal and any other BMJ products and to exploit all rights, as set out in our licence.

The Submitting Author accepts and understands that any supply made under these terms is made by BMJ to the Submitting Author unless you are acting as an employee on behalf of your employer or a postgraduate student of an affiliated institution which is paying any applicable article publishing charge ("APC") for Open Access articles. Where the Submitting Author wishes to make the Work available on an Open Access basis (and intends to pay the relevant APC), the terms of reuse of such Open Access shall be governed by a Creative Commons licence – details of these licences and which Creative Commons licence will apply to this Work are set out in our licence referred to above.

Other than as permitted in any relevant BMJ Author's Self Archiving Policies, I confirm this Work has not been accepted for publication elsewhere, is not being considered for publication elsewhere and does not duplicate material already published. I confirm all authors consent to publication of this Work and authorise the granting of this licence.

For peer review only

Supervised Treatment in Outpatients for Schizophrenia Plus (STOPS+): Protocol for a cluster randomised trial of a community-based intervention to improve treatment adherence and reduce the treatment gap for schizophrenia in Pakistan

Thomas A. Shepherd[1], Zia Ul Haq[2], Mian Muktar ul-Haq[3], Muhammad Firaz Khan[3], Adil Afridi[3], Lisa Dikomitis[1.6], Michelle E. Robinson[1], Martyn Lewis[1], Atif Rahman[4], Krysia Dziedzic[1], Umaima Saeed[2], Naila Riaz Awan[3] Christian D. Mallen[1, 5] & Saeed Farooq[1,5]

[1]School of Primary, Community and Social Care, Keele University, Staffordshire, ST5 5BG, UK

[2]Khyber Medical University, Phase V, Hayatabad, Peshawar, Khyber Pakhtunkhwa, 25100 Pakistan

[3]Lady Reading Hospital, Soekarno Rd, Pipal Mandi, Peshawar, Khyber Pakhtunkhwa 25000, Pakistan

[4]University of Liverpool, Foundation Building, Brownlow Hill, Liverpool, L69 7TX, UK

[5]Midlands Partnership Foundation Trust, Trust Headquarters, Trust Head Quarters Mellor House, Corporation St, Stafford ST16 3SR

[6] School of Medicine, Keele University Staffordshire, ST5 5BG, UK

Correspondence: Prof. Saeed Farooq, Faculty of Medicine and Health Sciences, Keele University, [1]School of Primary, Community and Social Care, Keele University, Staffordshire, ST5 5BG, UK

E mail: s.farooq@keele.ac.uk
Telephone: 01782 734973

Keywords: Psychosis, Low and middle Income Countries (LMIC), Drug Treatment, Directly Observed Therapy (DOTS)

Word Count: 5102

For peer review only - http://bmjopen.bmj.com/site/about/guidelines.xhtml

ABSTRACT

Introduction: There is a significant treatment gap, with only a few community-based services for people with schizophrenia in Low- and Middle-Income countries. Poor treatment adherence in schizophrenia is associated with poorer health outcomes, suicide attempts and death. We previously reported the effectiveness of STOPS (Supervised Treatment in Outpatients for Schizophrenia) for improving treatment adherence in patients with schizophrenia. However, STOPS was evaluated in a tertiary care setting with no primary care involvement, limiting its generalisability to the wider at-risk population. We aim to evaluate the effectiveness of STOPS+ in scaling up the primary care treatment of schizophrenia to a real-world setting.

Methods and analysis: The effectiveness of the STOPS+ intervention in improving the level of functioning and medication adherence in patients with schizophrenia in Pakistan will be evaluated using a cluster randomised controlled trial design. We aim to recruit 526 participants from 24 Primary Health Care Centres (PHCs) randomly allocated in 1:1 ratio to STOPS+ intervention and Enhanced Treatment as Usual (ETAU) arms. Participants will be followed-up for 12 months post recruitment. The sample size is estimated for two outcomes (i) The primary clinical outcome is level of functioning, measured using the Global Assessment of Functioning (GAF) scale, and (ii) the primary process outcome is adherence to treatment regimen measured using a validated measure. An intention-to-treat approach will be used for the primary analysis.

Ethics and Dissemination: Ethical approval has been obtained from Keele University Ethical Review Panel (ref: MH-190017) and Khyber Medical University Ethical Review Board (ref: DIR-KMU-EB/ST/000648). The results of the STOPS+ trial will be reported in peer reviewed journals and academic conferences and disseminated to local stakeholders and policy makers.

Trial Registration Number - ISRCTN: 93243890

For peer review only

STRENGTHS AND LIMITATIONS OF THIS STUDY

- This study aims to evaluate an approach that can be used for scaling up treatment for schizophrenia in resource poor settings and specifically addresses the gap in schizophrenia treatment.
- To the best of our knowledge this is first study that uses a DOTS (Directly Observed Therapy) approach translated from an infectious disorder i.e. Tuberculosis to a non-communicable disorder for scaling up the treatment for schizophrenia.
- The intervention involves primary care in treatment of schizophrenia in community, which is rarely done in LMIC.
- The trial is powered two primary outcomes (i) a clinical outcome measure – Global Assessment of Functioning and (ii) a process outcome measure – adherence to medication.
- The measurement of treatment adherence is based on a subjective measure i.e. reporting from patients and carers that cannot be objectively validated.

For peer review only

For peer review only - http://bmjopen.bmj.com/site/about/guidelines.xhtml

INTRODUCTION

Schizophrenia is one of the leading causes of years lived with disability, especially in low- and middle-income countries (LMICs) [1]. Existing evidence-based interventions for schizophrenia have been poorly implemented and most LMIC countries have large treatment gap for schizophrenia [2]. The treatment gap represents the disparity between the true prevalence of a disorder and the treated proportion of individuals diagnosed [3]. Pakistan has one of the largest treatment gaps for schizophrenia, at an estimated 96%, this is contrast to an estimated median of 32.2% globally and 69% in LMICs [4] [5].This treatment gap is attributed to a number of factors including poor treatment adherence with average non treatment adherence rates of about 50% in schizophrenia,[4] level of disability, a lack of primary care involvement and little to no access to treatment [5]. This is a particularly important factor in rural areas, where healthcare resources are often diminished in comparison to urban centres [6]. Whilst the cost of antipsychotic medication in Pakistan is relatively low, economic and logistical factors remain significant barriers to medication access [7] and the purchase of antipsychotic medication is often at the expense of other forms of essential out-of-pocket healthcare or even food [8].

Reducing the treatment gap for schizophrenia in LMIC is critical. Medication non-adherence in patients with schizophrenia is associated with 2.8 times increased risk in hospital admission [9]. The risk of death in patients who receive even minimal treatment is significantly reduced compared with patients receiving no treatment at all [10]. Furthermore, untreated psychosis can at least double the costs of managing chronic co-morbid physical health conditions, leading to an increased stress on an already overburdened healthcare system [11].

We previously reported a randomised controlled trial (RCT) of Supervised Treatment in Out-Patients for Schizophrenia (STOPS) in Peshawar, Pakistan [12]. STOPS was developed using the Medical Research Council framework for complex interventions [13] and was modelled on the Directly Observed Treatment Short-Course (DOTS) intervention to improve treatment adherence for people with Tuberculosis (TB). The DOTS approach involves a TB worker or a family member directly supervising the patient taking all medication and recording this [14]. DOTS is one of the most successful public health interventions and has been shown to be as highly effective in the community as it is in specialist clinics, which in addition to its accessibility and low cost, make it a highly appropriate intervention model for LMICs. Using the similar approach, STOPS intervention involved a family member of a person with schizophrenia observing and recording that medication had been taken. The family member is trained for this purpose. A previous evaluation of STOPS in a tertiary care setting showed that the STOPS significantly improved treatment adherence and functioning as well reducing patient symptoms [11].

The original STOPS intervention was evaluated in a tertiary care setting by mental health professionals, with no primary care involvement, limiting its scaling up and generalisability. Through a programme of qualitative work with key stakeholders in Pakistan, including patients, carers, healthcare professionals, researchers and local traditional healers, we adapted the STOPS model to STOPS+ with the aim of scaling up the intervention and involving primary care in the management of schizophrenia.

For peer review only - http://bmjopen.bmj.com/site/about/guidelines.xhtml

Here, we outline the protocol for evaluating the STOPS+ in a real-world setting using a cluster RCT design.

METHODS AND ANALYSIS

Objectives

Primary objective

The primary objective is to evaluate the clinical effectiveness of the STOPS+ intervention in improving medication adherence and functioning in patients with schizophrenia compared to enhanced treatment as usual (ETAU) in primary care in Pakistan.

Secondary objectives

i) To evaluate the cost effectiveness of the STOPS+ intervention compared to ETAU;
ii) To assess the effectiveness of STOPS+ in reducing family/caregiver burden and stigma in the community;
iii) To evaluate if STOPS+ leads to physical health improvement in patients with schizophrenia;
iv) To investigate the implementation of STOPS+ in a primary care setting and the acceptability of STOPS+ for service users and healthcare providers, assessing the impact of STOPS+ in the wider healthcare system.

Study setting

The intervention will be implemented and evaluated in the Peshawar district of Khyber Pakhtunkhwa (KP). KP is one of four provinces in Pakistan located in the North West region with an estimated population of 20.7 million. Health expenditure in Pakistan is about 1% of the Gross Domestic Product (GDP) with no separate provision for mental health services. The primary health care (PHC) facilities in KP are provided by a network of Basic Health Units (BHUs), Civil Dispensaries (CDs), and Rural Health Centres (RHCs). Typically, each PHC facility consists of a primary care physician, a Multipurpose PHC technician (MT) and a Lady Health Worker (LHW) [15]. Training PHC providers to manage mental health conditions and the incorporation of mental health services into primary care are both envisaged in current health policy but have not yet been fully realised [5].

Trial design

A cluster randomised controlled trial (RCT) will be used to investigate the efficacy of the STOPS+ intervention compared with Enhanced Treatment as Usual (ETAU) in people living with Schizophrenia. The cluster RCT design will help to evaluate the implementation of STOPS+ at the health care facility level and minimise the contamination of intervention and control arms.

The control: Enhanced Treatment as Usual (ETAU)

For peer review only - http://bmjopen.bmj.com/site/about/guidelines.xhtml

Typically, in Pakistan, treatment for schizophrenia consists of accessing hospital based psychiatric services with little to no involvement of PHC and a limited provision of medication from the hospital pharmacy. ETAU provided in the control clusters will include the treatment received by patients' routine health care setting, which includes treatment provided by a psychiatrist in the outpatient clinics of the psychiatry department of the local hospital and brief counselling about the treatment and outcome of the disorder. Patients may be admitted to an inpatient unit at the hospital. This will be enhanced in the control cluster PHCs with training provided to PHC physicians in WHO Mental Health Gap Action Programme (MhGAP) (https://www.who.int/mental_health/mhgap/en/t) in addition to the regular and reliable provision of psychotropic drugs at the PHC. The patients will be informed that the antipsychotic medication is available in the PHC centre and they can access the centre for supply of medication. The PHC physician will have the option to liaise with the treating psychiatrist, as provided in the MhGAP guidelines.

STOPS+ intervention

The STOPS+ intervention will consist of the ETAU plus the following components;

1. Supervision by a trained family member for dispensing and administering medication

A family member who is living with the patient for a minimum of 6 months prior to recruitment to the trial will be nominated by the patient. The family member will dispense medication, observe that is has been taken correctly and record this information on a simple sheet of paper designed and used in the previous STOPS trial [9]. Family members will be reminded to administer the medication by an automated text message system, which will also request a response once the medication has been taken correctly. The text message reminders will be sent every day for the first three months after starting the treatment. If the family member does not have access to a mobile phone, a handset and training in using it will be provided. A Multi-Purpose technician (MPT) from each PHC centre will provide training to family members for the storage and administration of the medication, recording when the medication has been taken correctly and the importance of maintaining the family dynamic.

2. Treatment for schizophrenia at PHC level supervised by mental health professionals

Ongoing management and monitoring of the patient's schizophrenia will take place at their PHC centre by the PHC physician, under the supervision of the treating psychiatrist with the help of the mobile technology platform SMART (Smartphone for Monitoring, Assessment and Remote Treatment). SMART will provide two-way communication between mental health care professionals. One month's supply of medication will be made available, via their PHC, to the family member. The automated messaging system will also remind the family member about collecting the following month's supply.

3. Monitoring the availability of essential psychotropic medication and their side effects

A kit to monitor the supply of medication will be used to monitor the availability and supply of psychotropic medication. This will consist of a) a cupboard dedicated specifically for the storage of psychotropic medication, b) a tracking sheet used to monitor the stock levels c) a simple checklist for monitoring side-effects of antipsychotics.

For peer review only - http://bmjopen.bmj.com/site/about/guidelines.xhtml

Assessment of barriers and facilitators of implementation of the intervention

The use of theory to inform the development of behaviour change interventions is strongly advocated by experts in the field. We have designed the process of implementation using theory developed for implementation and tested previously in UK Primary Care Practice [16,17]. We will evaluate the barriers and facilitators to implementation of the intervention using qualitative methods. We will hold at least four focus groups (with patients and carers, multipurpose technicians, traditional and faith healers and primary care clinicians) in which we will explore the barrier and facilitators to supervising the treatment of schizophrenia in the community, modification of the STOPS programme to STOPS+ for use in the community and the present status of schizophrenia care in the community

Patient and Public Involvement

STOPS+ is underpinned by culturally appropriate and context-bespoke patient and public community engagement and involvement at every stage of the project (intervention development, RCT and process evaluation). The STOPS+ intervention will be refined with input from the key stakeholder groups including patients, carers, health professionals, local councillors, community leaders such as religious leaders and community experts and policy makers. Small group meetings using local Jirgas (Jirga is traditional, local participatory and decision-making body which is well-established in Pathan culture and brings together local tribal, ethnic and religious leaders) will help in community engagement.

Primary outcome measure

In view of the pragmatic nature of the trial, we will use two distinct primary outcomes:
(i) The primary clinical outcome will be the level of functioning, measured using the Global Assessment of Functioning (GAF) scale.[18] GAF is a commonly used tool in schizophrenia, particularly for the pragmatic trials [19].
(ii) The primary process outcome will be adherence to treatment regimen. The adherence to treatment will be measured using a questionnaire adapted from Herz et al (2000).[20] This measure for assessing treatment adherence was successfully used in our previous STOPS study [9] .

Secondary outcomes:

The details of secondary outcomes and the standardised instruments for measuring these are given in Table 1. These instruments will measure secondary outcomes such as physical health outcomes, family/caregivers burden and internalised stigma.

Table 1. Description of outcome measures and instruments

Domains measured	Measure

For peer review only - http://bmjopen.bmj.com/site/about/guidelines.xhtml

	Sociodemographics	Age<br>Gender<br>Years in education<br>Marital status<br>Work status<br>Mini-Neuropsychiatric Interview (MINI) for diagnosis of schizophrenia [21]
	Physical health	Body Mass Index (BMI)<br>Blood Pressure<br>Waist Circumference
**Primary outcomes**		
	Level of functioning	Global Assessment of Functioning (GAF) [18]
	Level of adherence	Treatment adherence rating scale [20]
**Secondary outcomes**		
	Mental state and psychiatric symptoms	Brief Psychiatric Rating Scale [22]
	Caregivers' burden	Family Burden Scale [23]
	Perceived stigma	Internalised Stigma of Mental Illness [24]
	Side effects of antipsychotic medication	Glasgow Antipsychotic Side-Effects Scale [25]
	Cost of care	Client Service Receipt Inventory [26]
	Quality of life and cost effectiveness	EuroQol (EQ5D) [27]
	Illness severity, improvement and response to treatment	Clinical Global Impression Scale [28]
	Drug use	DAST Drug Screening Tool [29]
	Depression	Patient Health Questionnaire [30]
	Suicide Ideation	Suicide Behaviours Questionnaire Revised [31]

Eligibility criteria

Patients will be eligible for inclusion if they;

a) have a diagnosis of schizophrenia (F20.9) or schizoaffective disorder (F25.9) based on the International Classification of Disease 10 (ICD-10) criteria assessed using the Mini-International Neuropsychiatric Interview [21]

For peer review only - http://bmjopen.bmj.com/site/about/guidelines.xhtml

b) are aged between 17-65 years
c) do not meet the criteria for remission as defined by the Remission in Schizophrenia Working Group [32]
d) have capacity and are able to give informed consent
e) Have a family member who is also willing to participate in the trial and supervise the treatment after training by the research team. .

Exclusion criteria will be kept to a minimum to ensure that the study can examine the real world implementation of the STOPS+. The patients who can't participate in the study due to one of following reason will be excluded I) a serious or unstable medical illness, II) evidence of learning disability, III) severe drug dependence requiring treatment and/or detoxification IV) pregnant or breast-feeding.

Duration of treatment and follow-up:

Both groups will be followed up for 12 months after recruitment in the study. The follow up using face-to-face assessments will be carried out by trained research assistants in a health facility which is not part of participating PHCs to preserve the blindness of clusters. All of the outcomes will be measured at baseline, at 6 months and at 12 months by research assistants.

Participant Identification

Participants will be identified from two possible sources i) patients living in the area of the participating PHCs and presenting at psychiatry outpatient services of the hospitals in the area (ii) potentially eligible patients identified by the primary health care workers during community engagement or attending the PHCs. Eligible participants will be informed about the trial orally by the PHC staff and written information will be provided in the local language (Urdu). Those interested in taking part will be asked to give written informed consent. For participants who cannot read and write, witnessed oral consent and a thumb print in lieu of a signature will be used. The witness will not be a member of the research team. The procedure has been used in our previous trials in the area [33].

Randomisation and allocation concealment

Randomisation will occur at the PHC level, where PHCs will be allocated at a ratio of 1:1 to either deliver the STOPS+ intervention or ETAU. Twenty-four PHCs will be recruited to the study, 12 will be randomised to deliver ETAU and 12 will be randomised to deliver the STOPS+ programme. PHC randomisation will be stratified by urban/rural setting. Randomisation will be carried out by an independent statistician at the Khyber Medical University (KMU), Peshawar by using a remote computer generated random sequence. Randomisation and will be based on the list of primary care centres in Peshawar, provided by the health department of KP. The urban/rural classification is determined as per the Peshawar district council. It is not possible to blind study participants from their treatment arm allocation. However, the assessment team that conducts the baseline and follow up assessments (6 and 12 months) will be masked to the treatment arm of the cluster. The outcome measures will be administered by trained research assessment teams (outcome assessors), who are independent of the intervention team and the team involved in

For peer review only

consenting and screening. The outcome assessment will not be carried out at any participating PHC. The assessment teams will be told that they are evaluating two interventions and that there is genuine equipoise about which one is better. Study participants will be instructed to not discuss the PHC they are attending or the treatment details. During all assessments, the primary outcome measures (treatment adherence scale and GAF) will be completed first to minimise the risk of bias in the event of unmasking and, if it occurs, the point of unmasking will be recorded. Sensitivity analyses will be carried out to assess the effect of unmasking on the primary outcomes. The data linking each PHC with treatment allocation status is kept separate from the outcome dataset until the time of the final analysis. The trial statistician will be blind to allocation status.

Screening, Data collection and follow up

After identification of potentially eligible patients, they will first be orally informed about the trial by one of the health workers in the local Pushto language (MT in the community or a health professional in one of the health facilities mentioned above). They will be asked whether they agree that a member of the research team will provide them with further information about the research. If permission is given, a research assistant will meet with the patient and will request informed consent. The informed consent for participation in the study will be taken within one week of the first contact. Participants will be free to decline to participate or withdraw at any time without their routine health care being affected. Before taking part in any interviews, oral and written information about the study and its purpose will be provided to respondents in the local language.

After obtaining informed consent the research assistant will decide whether the patient meets the inclusion criteria and have a family member who is willing to supervise the treatment as specified in the STOPS+ procedures. If the patient is not eligible from the screening assessment the reason for ineligibility will be explained to the patient and they will be signposted for further advice and to continue to receive treatment at their routine health facility. Eligible patients will be provided with a further participant information sheet about the trial. Additionally, for those in the intervention arm, the nominated relative will receive a participant information sheet about the trial and about the tasks required in supervising the treatment If nominated family member is available with the patient, this information will be provided at the same time. If the family member is not available, patients and the family member will be asked to visit the health facility again within one week. The patient and the relative will each be given the opportunity to ask any questions about the trial. The information sheets are provided in Urdu. For illiterate patients the research assistants will explain the same information in local Pushto language or in Urdu. Following the informed consent, the baseline data will be collected.

Sample size calculation

A mean difference of 6 on the primary clinical outcome measure (GAF) with a pooled study standard deviation of 15 was observed in our previous trial in the same setting [12]. A recent indicated minimum clinically important difference for the GAF in schizophrenia patients has been given as a mean difference of 4) [34]. A total sample size of 526 i.e. 263 patients in each arm (recruited across 24 PHCs, with an average cluster size of 22 patients) has over 90% power to detect a mean difference of 6 and 85-90% power to detect a more modest

For peer review only - http://bmjopen.bmj.com/site/about/guidelines.xhtml

mean difference of 4 in the GAF given a two-tailed significance level of 0.05 and based on the following parameter assumptions: i) baseline-outcome correlation of 0.5, repeated measures correlation of 0.7 [35] and loss to follow up of 20% (patient-level parameters); ii) intracluster correlation coefficient of 0.01 [36] and coefficient of variation in cluster sizes of 0.65 (PHC-level parameters) [37]. The primary endpoint for the GAF is the 'average' GAF across 6 and 12 months follow up.

This sample size has 90% power to detect at least a 20% absolute difference in the treatment adherence rate between the intervention arm compared to the ETAU arm at 6 and/or 12 months follow up based on two-tailed alpha of 0.025 (to account for multiple testing in respect of 6 and 12 month testing). This is based on an observed ≈20% higher level of adherence within the intervention compared to ETAU arms in the original STOPS trial at 3 and 12 months follow up,[11] and further assuming the design effect PHC-level parameters as noted in (ii) above. Thus, the study is adequately powered to detect significant differences (at the level of 2-tailed alpha of 0.05 in each case) across the two distinct primary outcomes: i) the primary clinical outcome (GAF) and ii) the primary process outcome (medication adherence).

Trial management and monitoring

The STOPS+ trial will be monitored in line with the protocol and the trial standard operating procedures. An independent Trial Steering Committee (TSC) which will have service user representatives will monitor trial progress. The TSC is empowered to independently review the ethical and data management procedures. A Data and Safety Monitoring Board (DSMB) will be convened to ensure the safety of participants and the integrity of the data. The PHCs in the trial will receive training around safety reporting and additionally, participants and their family members will be informed of how to report concerns and safety issues, whilst they are participating in the trial. The Project Management Committee (PMC) will be responsible for setting up the trial, ongoing management and monitoring, promotion of the study, training and the interpretation of the results.

Safety and Adverse events reporting

All adverse events and serious adverse events (SAEs) that are reported by the participant, family member or identified by the trial team or PHC staff members during the trial will be recorded and reported following trial safety reporting procedures. Related and unexpected SAEs will be reported to the local Research Ethics Committee (REC), DSMB and TSC within 15 days. The DSMB will review all safety data and make any decisions regarding early termination of the study in line with their respective terms of reference.

Data management

All data will be managed in line with the protocol and will be stored securely following the FKMU and Keele University standard policy on data storage and confidentiality. Individual participant data will be pseudonymised through the use of a unique study ID and will be stored securely and separately from any identifiable data. Access to data will be restricted to members of the research team. Outcome data will be entered into an electronic database

For peer review only - http://bmjopen.bmj.com/site/about/guidelines.xhtml

which will be stored on an encrypted secure network at KMU requiring a password to access. Data entry will be quality checked and coded using standard processes.

Statistical analysis

Statistical analysis will be performed blind to cluster allocation. Primary analysis will be on an intention-to-treat (ITT) basis, with all participants analysed in their cluster. Between-group differences for both primary outcome measures (GAF and medication adherence) will be analysed using longitudinal mixed models that accommodate clustering at the (upper/hierarchical) practice level, as well as repeated outcome measurements across 3 time points (baseline, 6 and 12 months) per participant. i.e. linear/logistic mixed models for numerical and categorical outcomes, respectively, including PHC and participant as random factors . These models will be used to quantify the absolute between group difference in the primary clinical outcome (GAF) and odds ratio (and extracted absolute difference) for the primary process outcome (% adherence) – using linear and logistic models, respectively. For the GAF, the primary endpoint is the 'average' GAF over 6 and 12 months using available and modelled data to estimate the combined summary average mean difference in GAF between treatment arms over the 6 and 12 months timeline; statistical significance in this case being given against two-tailed p=0.05. Though the 'average' is the single primary endpoint for the GAF, we will also evaluate the treatment-by-time interaction to give estimates of between-arm mean differences at the individual 6 and 12 months follow-up time points (secondary endpoints).

For medication adherence, between-group comparison at both 6 and 12 months are primary (derived through treatment-by-time interaction within the generalised mixed model) – hence, the requirement to take into account the multiple-testing by assessing significance against a more conservative two-tailed p=0.025. Estimates of between-group mean differences will be given including 95% confidence intervals and p-values. The mixed models (detailed above) will be adjusted for age, gender, baseline GAF score and corresponding baseline score of the outcome being measured as individual-patient level covariates, PHC-level characteristics (PHC size), stratification variable (rural/urban) and a random effect for the PHC. Mixed model analysis fulfils the ITT principle with missing data being accounted for under the missing at random (MAR) assumption.

Cost effectiveness

The cost of STOPS+ will be evaluated against ETAU using the Service Receipt Inventory (SRI), [38] which has been used in previous trials in Pakistan [21]. This will include the cost of primary and outpatient care visits, inpatient admissions, drug regimens, diagnostic tests, travel time, transport costs and lost days from work. Costs will be subsequently linked to outcomes in order to establish whether STOPS+ represents a cost-effective use of resources (e.g. whether the additional cost of supervision by the relative are offset by the reduced hospital care and/or improvements in the clinical outcome – GAF and QALY). Cost-effectiveness planes and acceptability curves will evaluate the uncertainty in estimates as well as the probability of STOPS+ being cost-effective across a range of willingness-to-pay thresholds. Adjusted analysis will be carried to accommodate clustering of PHCs.

ETHICS AND DISSEMINATION

For peer review only - http://bmjopen.bmj.com/site/about/guidelines.xhtml

Ethical approval to conduct this trial have been obtained from Keele University Ethical review Panel (reference: MH-190017) and Khyber Medical University Ethical Review Board (ref: DIR-KMU-EB/ST/000648). Subsequently, recruitment began on the 1st of November 2019. Results will be disseminated through peer reviewed journals and academic conferences. The trial is being conducted in accordance with the International Conference on Harmonisation/Good Clinical Practice (ICHGCP) Guidelines (1996) which are consistent with principles of Declaration of Helsinki (1996) Ethical Principles for Medical Research Involving Human Subjects.

Process evaluation

Patients and relatives, primary care physicians and multipurpose technician from participating PHCs in both arms will be invited to take part in a semi-structured interview to assess the acceptability of intervention and its implementation in the health system [39]. These interviews will last approximately 30-40 minutes and will be audio recorded with permission. A minimum of 20 individual semi-structured interviews [40] will be conducted (continuing until data saturation is reached)[41,42] with patients and their relatives to explore a) the acceptability of the STOPS+ intervention; b) the experience/burden of the training and support for the patient's family member; c) barriers and facilitators to medication adherence and overall satisfaction with the intervention and trial participation. A minimum of 10 interviews will be conducted with PHC physicians. In addition, we will conduct interviews with at least 5 decision-makers with responsibilities for developing or implementing health policy in the directorate of health in order to capture their perceptions about the feasibility, the benefits, challenges and acceptability of STOPS+ in the health system. Participants for these interviews will be purposively sampled to stratify by gender and urban/rural setting. A semi-structured topic guide will be used to facilitate interviews. Informed consent will be obtained prior to the start of the interview and will include consent for the use of direct pseudonymised quotes.

An inductive, exploratory framework will be adopted using thematic analysis based on the principles of grounded theory.[43,44] A sample of early transcripts will be independently coded by the research team and a coding framework agreed upon. This framework will then be applied to subsequent coding. Coded data will be analysed independently by qualitative study leads at Keele and KMU to develop categories and themes to be discussed in wider research team meetings. Constant comparison will be used to explore connections within and across transcripts and codes, highlighting consistencies and variation [45]. Analysis will follow an iterative process, with emergent findings used to further refine topic guides for subsequent interviews.

DISCUSSION

People suffering from schizophrenia represent one of the most disadvantaged groups in low- and middle-income countries facing a wide mental health treatment gap. A number of psychosocial interventions have been evaluated in RCTs and there is consensus that the treatment of schizophrenia should combine anti-psychotic medication and psychosocial

For peer review only

interventions [46]. However there is limited evidence how these interventions can be implemented in real world settings.

The proposed study adopts an implementation approach addressing systemic barriers to reduce treatment gap for schizophrenia in the community. The STOPS+ intervention aims to address three major implementation barriers (i) improving supply of antipsychotic medication at primary care level (ii) involving primary care workers in the treatment of schizophrenia (iii) improving treatment adherence by involving family members in supervising treatment. The STOPS+ approach therefore goes beyond most psychosocial interventions evaluated in treatment of schizophrenia in LMIC countries [47, 48] in addressing the health system, community and service users related factors to improve access to treatment.
A unique aspect of the trial is that it is powered for both process and clinical outcomes. These two primary outcomes are: (i) a clinical outcome measure – Global Assessment of Functioning and (ii) a process outcome measure – adherence to medication. This combined with a process evaluation carried out after completion of the cluster RCT will provide much needed evidence on the implementation of community-based interventions for schizophrenia in LMIC countries settings. The WHO Mental Health Action Plan (2013-2020) requires that there is an absolute increase of 20% in service coverage for severe mental disorders [48]. The results of the trial may help to understand the processes and implementation strategies to achieve that goal and could provide an effective public health intervention for schizophrenia [49].

A major strength of the STOPS intervention is that it is based on an infectious disease model that has been successfully used in improving treatment adherence for Tuberculosis i.e. DOTS (http://www.searo.who.int/tb/topics/what_dots/en/) as a public health intervention. The DOTS (Directly Observed Treatment Short course) model is currently being used in most LMIC countries, including the settings of this trial. This can potentially help in integration of the care for a non-communicable chronic disorder like schizophrenia in the public health systems of LMIC countries.

Competing interests statement: SF in the past 3 years has received honoraria and speaker's fees from Otsuka, Lundbeck and Sunovian pharma and also research funding from Sunovian Pharmaceutical. MMuH and MFK received funding from different pharmaceuticals to attend scientific meetings.

Acknowledgements: Krysia Dziedzic is an NIHR Senior Investigator. The views expressed in this paper are those of the authors and not necessarily those of the NHS, NIHR, or the Department of Health and Social Care.

For peer review only - http://bmjopen.bmj.com/site/about/guidelines.xhtml

References

1 GBD 2017 Disease and Injury Incidence and Prevalence Collaborators. Global, regional, and national incidence, prevalence, and years lived with disability for 354 diseases and injuries for 195 countries and territories, 1990–2017: a systematic analysis for the Global Burden of Disease Study 2017. *The Lancet. 2018;**392**(10159)*:1789-1858

2 Charlson FJ, Ferrari AJ, Santomauro DJ, et al. Global Epidemiology and Burden of Schizophrenia: Findings From the Global Burden of Disease Study 2016. *Schizophrenia Bulletin* 2018;**44**(6):1195-1203.

3 Patel V, Maj M, De Silva MJ, Koschorke M & Prince M. Estimating the treatment gap for mental disorders: a WPA survey. *World Psychiatry* 2010:9(3) 169-176

4 Lora A, Kohn R, Levav I, et al. Service availability and utilization and treatment gap for schizophrenic disorders: a survey in 50 low- and middle-income countries. *Bull World Health Organ* 2012;**90**(1):47-54B.

5 De Silva MJ, Lee L, Fuhr DC, Rathod S, Chisholm D, Schellenberg J & Patel V. Estimating coverage of mental health programs: a systematic review. *International Journal of Epidemiology* 2014;43(2) 341-353.

6 Kagstrom A, Alexova A, Tuskova E, Csajbók Z, Schomerus G, Formanek T, Mladá K, Winkler P, Cermakova P. The treatment gap for mental disorders and associated factors in the Czech Republic. *European Psychiatry* 2019;59,37-43.

7 Large M, Farooq S, Nielssen O, et al. Relationship between gross domestic product and duration of untreated psychosis in low- and middle-income countries. *Br J Psychiatry* 2008;**193**(4):272-278.

8 Farooq S, Burns J, Sumathipala A, et al. A Global Mental Health Fund for the treatment of Serious Mental Illness in Low Income and Middle Income Countries. *The Lancet Psychiatry 2016;***3**(6):182-183.

9 Weiden PJ and Olfson M. Cost of relapse in schizophrenia. *Schizophr Bull* 1995;**21**(3):419-429.

10 Farooq S, Large M, Nielssen O, et al. The relationship between the duration of untreated psychosis and outcome in low-and-middle income countries: a systematic review and meta analysis. *Schizophr Res* 2009;**109**(1-3):15-23.

11 McDaid D and Park A-L. Counting all the costs: the economic impact of comorbidity. *Karger Publishers* 2014.

12 Farooq S, Nazar Z, Irfan M, et al. Schizophrenia medication adherence in a resource-poor setting: randomised controlled trial of supervised treatment in out-patients for schizophrenia (STOPS). *Br J Psychiatry* 2011;**199**(6):467-472.

For peer review only

13 Campbell M, Fitzpatrick R, Haines A, et al. Framework for design and evaluation of complex interventions to improve health. *BMJ* 2000;**321**(7262):694-696.

14 World Health Organization. Communicable Diseases Cluster. (1999). What is DOTS? : a guide to understanding the WHO-recommended TB control strategy known as DOTS. World Health Organization.

15 Government of Khyber Pakhtunkhwa. Mental Health And Psychosocial Support: Strategy And 5-Year Multi-Sectoral Action Plan For Khyber Pakhtunkhwa (2017-2022). *Government of Khyber Pakhtunkhwa* 2017.

16 Grol R, Wensing M, Eccles M: Improving Patient Care - The Implementation of Change in Clinical Practice. 2004, London: Elsevier

17 Michie S, van Stralen MM, West R: The behaviour change wheel: a new method for characterising and designing behaviour change interventions. Implement Sci. 2011, 6: 42-10.1186/1748-5908-6-42.

18 American Psychiatric Association. DSM-IV-TR: Diagnostic and statistical manual of mental disorders, text revision. Washington, DC: American Psychiatric Association 2000;**75**.

19 Crawford MJ, Killaspy H, Barnes TRE, et al. Group art therapy as an adjunctive treatment for people with schizophrenia: multicentre pragmatic randomised trial. *BMJ* 2012;344:e846

20 Herz MI, Lamberti S, Mintz J, et al. A program for relapse prevention in schizophrenia. A controlled study. *Arch Gen Psychiatry* 2000;57:277–283.

21 Sheehan DV, Lecrubier Y, Sheehan KH, et al. The Mini-International Neuropsychiatric Interview (M.I.N.I.): the development and validation of a structured diagnostic psychiatric interview for DSM-IV and ICD-10. *J Clin Psychiatry* 1998;**59**(Suppl 20):22-33.

22 Overall, JE, & Gorham, DR. The Brief Psychiatric Rating Scale. *Psychological Reports* 1962;10(3):799–812.

23 Pai S & Kapur RL. The burden on the family of a psychiatric patient: development of an interview schedule. *Br J Psychiatry* 1981;**138**:332-335.

24 Lysaker PH, Davis LW, Warma DM, Strasburger A & Beattie N. Stigma, social function and symptoms in schizophrenia and schizoaffective disorder: associations across 6 months**.** *Psychiatry Research* 2007;14:89-95.

25 Waddell L, Taylor M. A new self-rating scale for detecting atypical or second-generation antipsychotic side effects. *Journal Psychopharmacology* 2008; 22:238

26 Chisholm D, Knapp MR, Knudsen HC, Amaddeo F, Gaite L, van Wijngaarden B. Client Socio-Demographic and Service Receipt Inventory--European Version: development of an instrument for international research. EPSILON Study 5. European Psychiatric Services:

For peer review only - http://bmjopen.bmj.com/site/about/guidelines.xhtml

Inputs Linked to Outcome Domains and Needs.
Br J Psychiatry Suppl. 2000;(39):28-33.

27 The EuroQol Group (1990). EuroQol-a new facility for the measurement of health-related quality of life. *Health Policy* 1990;16(3):199-208.

28 Guy W (ed). ECDEU Assessment Manual for Psychopharmacology. Rockville, MD: US Department of Heath,Education, and Welfare Public Health Service Alcohol, Drug Abuse, and Mental Health Administration, 1976 Busner J, Targum SD. The clinical global impressions scale: applying a research tool in clinical practice. *Psychiatry (Edgmont)* 2007;4(7):28-37

29 Skinner HA. The Drug Abuse Screening Test. *Addictive Behavior* 1982;*7*(4): 363–371.

30 Kroenke K, Spintzer RL, Williams JB. The PHQ-9: validity of a brief depression severity measure. Journal of General Internal Medicine 2001;16(9):606-613

31 Osman A, Bagge CL, Gutierrez PM, Konick LC, Kopper BA, Barrios FX., The Suicide Behaviours Questionnaire-Revised (SBQ-R): Validation with clinical and nonclinical samples. *Assessment*. 2001 (5):443-54

32 Andreasen NC, Carpenter WT Jr, Kane JM, et al. Remission in schizophrenia: proposed criteria and rationale for consensus. *Am J Psychiatry* 2005;**162**(3):441-449.

33 Rahman A, Hamdani SU, Awan NR, et al. (2016). Effect of a Multicomponent Behavioural Intervention in Adults Impaired by Psychological Distress in a Conflict-Affected Area of Pakistan: A Randomized Clinical Trial. *JAMA* 2016;316(24):2609-2617.

34 Amri I, Millier A, Toumi M. PMH2 - Minimum Clinically Important Difference in the Global Assessment Functioning in Patients with Schizophrenia. *Value in Health* 2014;17:A765–A766.

35 Vickers AJ. How many repeated measures in repeated measures designs? Statistical issues for comparative trials. *BMC Med Res Methodol* 2003;**3**:22.

36 Adams G, Gulliford MC, Ukoumunne OC, et al. Patterns of intra-cluster correlation from primary care research to inform study design and analysis. *J Clin Epidemiol* 2004;**57**(8):785-94.

37 Eldridge SM, Ashby D and Kerry S. Sample size for cluster randomized trials: effect of coefficient of variation of cluster size and analysis method. *Int J Epidemiol* 2006;35(5):1292-300.

38 Chisholm D, Knapp MR, Knudsen HC, et al. Client socio-demographic and service receipt inventory--european version: development of an instrument for international research. EPSILON study 5. European psychiatric services: inputs linked to outcome domains and needs. *Br J Psychiatry Suppl* 2000;39:s28–33.

39 O'Cathain A. A practical guide to using qualitative research with randomized controlled trials. Oxford University Press 2018.

For peer review only - http://bmjopen.bmj.com/site/about/guidelines.xhtml

40 Malterud K, Siersma VD and Guassora AD. Sample size in qualitative interview studies: guided by information power. *Qualitative health research* 2016;26(13):1753-1760.

41 Saunders B, Sim J, Kingstone T, et al 2018. Saturation in qualitative research: exploring its conceptualization and operationalization. *Quality & quantity* 2018;52(4):1893-1907.

42 Sim J, Saunders B, Waterfield J, et al. Can sample size in qualitative research be determined a priori?. *International Journal of Social Research Methodology 2018;*21(5):619-634.

43 Glaser BG and Strauss AL. Discovery of grounded theory: Strategies for qualitative research. Routledge 2017.

44 Braun V, Clarke V, Hayfield N, et al. Thematic analysis. Handbook of research methods in health social sciences. Singapore: Springer 2018:1-18.

45 Nowell LS, Norris JM, White DE, et al. Thematic analysis: Striving to meet the trustworthiness criteria. *International Journal of Qualitative Methods* 2017;16(1): p.1609406917733847.

46 Asher L, Patel V, De Sliva MJ. Community-based psychosocial interventions for people with schizophrenia in low and middle-income countries: systematic review and meta-analysis. *BMC Psychiatry* 2017;**17**:355.

47 Xu DR, Xiao S, He H, et al. Lay health supporters aided by mobile text messaging to improve adherence, symptoms, and functioning among people with schizophrenia in a resource-poor community in rural China (LEAN): A randomized controlled trial. *PLoS Med* 2019;**16**(4):e1002785.

48 Kaddick H, Horney B, McKenzie J, Tilley H. Investing in mental health in low and middle income countries. Overseas Development Institute, London 2016.

49 Farooq, S. Public health and physician focused strategies to improve medication adherence in psychotic disorders. *World Psychiatry* 2013;12(3):238-239.

Authors' contributions: TAS wrote first draft. All authors contributed to the conceptualisation and the design of the study. SF obtained the funding for the study, conceived and developed the intervention and study design, and led creation of the team. MFK, ZuH, MMuH, AA, NRA and US secured the study sites and contributed to design and development of intervention, community engagement and qualitative studies leading to modification of intervention. LD contributed to design and led qualitative studies for intervention development and process evaluation. US, TAS, ZuH and MER contributed to writing ethics applications and obtaining the ethics approvals. ML contributed significantly to the study design, analysis plan and economic evaluation part of the protocol. AR,LD, KD, CDM provided critical review and contributed to the design of the study. All authors read and revised the initial manuscript and approved the final version.

For peer review only - http://bmjopen.bmj.com/site/about/guidelines.xhtml

Funding statement – This work is supported by the Medical Research Council UK, as part of their Global Alliance for Chronic Disease programme grant number MR/S00243X/1.

For peer review only

STANDARD PROTOCOL ITEMS: RECOMMENDATIONS FOR INTERVENTIONAL TRIALS

SPIRIT 2013 Checklist: Recommended items to address in a clinical trial protocol and related documents*

Section/item	Item No	Description	Addressed on page number
**Administrative information**			
Title	1	Descriptive title identifying the study design, population, interventions, and, if applicable, trial acronym	______8_______
Trial registration	2a	Trial identifier and registry name. If not yet registered, name of intended registry	______39_______
	2b	All items from the World Health Organization Trial Registration Data Set	_______*______
Protocol version	3	Date and version identifier	_______1______
Funding	4	Sources and types of financial, material, and other support	_______5______
Roles and responsibilities	5a	Names, affiliations, and roles of protocol contributors	_______5,6,7____
	5b	Name and contact information for the trial sponsor	_______5*______
	5c	Role of study sponsor and funders, if any, in study design; collection, management, analysis, and interpretation of data; writing of the report; and the decision to submit the report for publication, including whether they will have ultimate authority over any of these activities	______na_______
	5d	Composition, roles, and responsibilities of the coordinating centre, steering committee, endpoint adjudication committee, data management team, and other individuals or groups overseeing the trial, if applicable (see Item 21a for data monitoring committee)	______na_______

For peer review only

Introduction

Background and rationale	6a	Description of research question and justification for undertaking the trial, including summary of relevant studies (published and unpublished) examining benefits and harms for each intervention	_______10,11____
	6b	Explanation for choice of comparators	________11______
Objectives	7	Specific objectives or hypotheses	_______12_______
Trial design	8	Description of trial design including type of trial (eg, parallel group, crossover, factorial, single group), allocation ratio, and framework (eg, superiority, equivalence, noninferiority, exploratory)	________?______

Methods: Participants, interventions, and outcomes

Study setting	9	Description of study settings (eg, community clinic, academic hospital) and list of countries where data will be collected. Reference to where list of study sites can be obtained	_______17,18____
Eligibility criteria	10	Inclusion and exclusion criteria for participants. If applicable, eligibility criteria for study centres and individuals who will perform the interventions (eg, surgeons, psychotherapists)	_______18_______
Interventions	11a	Interventions for each group with sufficient detail to allow replication, including how and when they will be administered	_____14,15,16,17_
	11b	Criteria for discontinuing or modifying allocated interventions for a given trial participant (eg, drug dose change in response to harms, participant request, or improving/worsening disease)	_____26_______
	11c	Strategies to improve adherence to intervention protocols, and any procedures for monitoring adherence (eg, drug tablet return, laboratory tests)	_______16, 34____
	11d	Relevant concomitant care and interventions that are permitted or prohibited during the trial	_______26_______
Outcomes	12	Primary, secondary, and other outcomes, including the specific measurement variable (eg, systolic blood pressure), analysis metric (eg, change from baseline, final value, time to event), method of aggregation (eg, median, proportion), and time point for each outcome. Explanation of the clinical relevance of chosen efficacy and harm outcomes is strongly recommended	12,13,21,22,23,24
Participant timeline	13	Time schedule of enrolment, interventions (including any run-ins and washouts), assessments, and visits for participants. A schematic diagram is highly recommended (see Figure)	_______9_______

For peer review only - http://bmjopen.bmj.com/site/about/guidelines.xhtml

Sample size	14	Estimated number of participants needed to achieve study objectives and how it was determined, including clinical and statistical assumptions supporting any sample size calculations	___26,27_______
Recruitment	15	Strategies for achieving adequate participant enrolment to reach target sample size	______27_______

Methods: Assignment of interventions (for controlled trials)

Allocation:

Sequence generation	16a	Method of generating the allocation sequence (eg, computer-generated random numbers), and list of any factors for stratification. To reduce predictability of a random sequence, details of any planned restriction (eg, blocking) should be provided in a separate document that is unavailable to those who enrol participants or assign interventions	______19_______
Allocation concealment mechanism	16b	Mechanism of implementing the allocation sequence (eg, central telephone; sequentially numbered, opaque, sealed envelopes), describing any steps to conceal the sequence until interventions are assigned	_______8______
Implementation	16c	Who will generate the allocation sequence, who will enrol participants, and who will assign participants to interventions	_______19______
Blinding (masking)	17a	Who will be blinded after assignment to interventions (eg, trial participants, care providers, outcome assessors, data analysts), and how	____19,20______
	17b	If blinded, circumstances under which unblinding is permissible, and procedure for revealing a participant's allocated intervention during the trial	_____________

Methods: Data collection, management, and analysis

Data collection methods	18a	Plans for assessment and collection of outcome, baseline, and other trial data, including any related processes to promote data quality (eg, duplicate measurements, training of assessors) and a description of study instruments (eg, questionnaires, laboratory tests) along with their reliability and validity, if known. Reference to where data collection forms can be found, if not in the protocol	__21,22,23,24___
	18b	Plans to promote participant retention and complete follow-up, including list of any outcome data to be collected for participants who discontinue or deviate from intervention protocols	______na_______

Data management	19	Plans for data entry, coding, security, and storage, including any related processes to promote data quality (eg, double data entry; range checks for data values). Reference to where details of data management procedures can be found, if not in the protocol	___32,33,34_____
Statistical methods	20a	Statistical methods for analysing primary and secondary outcomes. Reference to where other details of the statistical analysis plan can be found, if not in the protocol	______28,29____
	20b	Methods for any additional analyses (eg, subgroup and adjusted analyses)	________29,30___
	20c	Definition of analysis population relating to protocol non-adherence (eg, as randomised analysis), and any statistical methods to handle missing data (eg, multiple imputation)	______29_______
**Methods: Monitoring**			
Data monitoring	21a	Composition of data monitoring committee (DMC); summary of its role and reporting structure; statement of whether it is independent from the sponsor and competing interests; and reference to where further details about its charter can be found, if not in the protocol. Alternatively, an explanation of why a DMC is not needed	______34_______
	21b	Description of any interim analyses and stopping guidelines, including who will have access to these interim results and make the final decision to terminate the trial	______30_______
Harms	22	Plans for collecting, assessing, reporting, and managing solicited and spontaneously reported adverse events and other unintended effects of trial interventions or trial conduct	___35,36,45_____
Auditing	23	Frequency and procedures for auditing trial conduct, if any, and whether the process will be independent from investigators and the sponsor	___33,34,35,36__
**Ethics and dissemination**			
Research ethics approval	24	Plans for seeking research ethics committee/institutional review board (REC/IRB) approval	______39_______
Protocol amendments	25	Plans for communicating important protocol modifications (eg, changes to eligibility criteria, outcomes, analyses) to relevant parties (eg, investigators, REC/IRBs, trial participants, trial registries, journals, regulators)	______na_______

For peer review only - http://bmjopen.bmj.com/site/about/guidelines.xhtml

Consent or assent	26a	Who will obtain informed consent or assent from potential trial participants or authorised surrogates, and how (see Item 32)	_______20________
	26b	Additional consent provisions for collection and use of participant data and biological specimens in ancillary studies, if applicable	_______na________
Confidentiality	27	How personal information about potential and enrolled participants will be collected, shared, and maintained in order to protect confidentiality before, during, and after the trial	_____31,32,33_____
Declaration of interests	28	Financial and other competing interests for principal investigators for the overall trial and each study site	_______39________
Access to data	29	Statement of who will have access to the final trial dataset, and disclosure of contractual agreements that limit such access for investigators	_______40________
Ancillary and post-trial care	30	Provisions, if any, for ancillary and post-trial care, and for compensation to those who suffer harm from trial participation	_________39_______
Dissemination policy	31a	Plans for investigators and sponsor to communicate trial results to participants, healthcare professionals, the public, and other relevant groups (eg, via publication, reporting in results databases, or other data sharing arrangements), including any publication restrictions	_____40,41_______
	31b	Authorship eligibility guidelines and any intended use of professional writers	_________*_______
	31c	Plans, if any, for granting public access to the full protocol, participant-level dataset, and statistical code	_________*_______
**Appendices**			
Informed consent materials	32	Model consent form and other related documentation given to participants and authorised surrogates	_______*________
Biological specimens	33	Plans for collection, laboratory evaluation, and storage of biological specimens for genetic or molecular analysis in the current trial and for future use in ancillary studies, if applicable	_______na________

*It is strongly recommended that this checklist be read in conjunction with the SPIRIT 2013 Explanation & Elaboration for important clarification on the items. Amendments to the protocol should be tracked and dated. The SPIRIT checklist is copyrighted by the SPIRIT Group under the Creative Commons "Attribution-NonCommercial-NoDerivs 3.0 Unported" license.
